# KLF1 Promotes Cardiomyocyte Proliferation and Heart Regeneration Through Regulation of Wnt/β‐Catenin Signaling Pathway

**DOI:** 10.1002/advs.202413964

**Published:** 2025-03-27

**Authors:** Yanglin Hao, Xi Zhang, Shuan Ran, Yuan Li, Weicong Ye, Song Wang, Xiaohan Li, Zilong Luo, Jiulu Zhao, Junjie Zong, Kexiao Zheng, Ran Li, Han Zhang, Longyong Lai, Pinyan Huang, Zifeng Zou, Wang Zhan, Zhang Yue, Jie Wu, Jiahong Xia

**Affiliations:** ^1^ Department of Cardiovascular Surgery, Union Hospital, Tongji Medical College Huazhong University of Science and Technology Wuhan 430022 China

**Keywords:** cardiomyocyte proliferation and heart regeneration, heart failure, KLF1, Wnt/β‐catenin signaling pathway

## Abstract

Innovative therapeutic approaches for heart failure, a leading cause of mortality worldwide, are urgently needed. In this study, the important role of Krüppel‐like factor 1 (KLF1) in cardiomyocyte proliferation and heart regeneration is explored, and revealed its ability to regulate the Wnt/β‐catenin signaling pathway as well as exploring a feasible strategy to target KLF1 for the treatment of heart failure. Postnatally, a marked decrease in KLF1 expression occurred almost simultaneously with a reduction in myocardial regenerative capacity. Through comprehensive in vivo and in vitro studies, it is demonstrated that in neonatal and adult mice, KLF1 overexpression significantly increased cardiomyocyte proliferation and promoted myocardial repair following infarction, whereas KLF1 knockout abolished these effects. Mechanistically, through RNA sequencing (RNA‐seq) and ATAC sequencing (ATAC‐seq) analyses, it is revealed that the promotion of cardiomyocyte proliferation by KLF1 is associated with the Wnt/β‐catenin signaling pathway, mitochondrial function, and fatty acid metabolism. These findings highlight the important role of KLF1 in cardiomyocyte proliferation and heart regeneration, which provides novel insights into therapeutic targets for heart failure.

## Graphical abstract

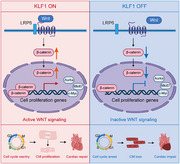

## 1 Introduction

Heart disease, including hypertrophic cardiomyopathy, ischemic heart disease and hypertension, remains a leading cause of mortality worldwide^[^ ^1^ ^]^ and frequently results in cardiac injury associated with heart failure because of the limited regenerative capacity of the adult mammalian heart.^[^ ^2^ ^]^ Following cardiac injury, damaged tissue is typically replaced by fibrotic scars rather than functional tissue, thus severely impairing heart recovery. In contrast, the neonatal heart has remarkable regenerative potential and is able to fully recover from significant injuries such as myocardial infarction (MI) and apex resection.^[^ ^3^, ^4^ ^]^ However, the regenerative capacity of cardiomyocytes decreases rapidly with age.^[^ ^5^ ^]^ Understanding the mechanisms of cardiomyocyte proliferation and identifying the factors associated with decreased regenerative ability are critical for developing effective strategies to treat heart failure resulting from myocardial damage.

The Wnt/β‐catenin signaling pathway is a fundamental cellular communication system with extensive involvement in the progression and treatment of cardiac disease.^[^ ^6^ ^]^ Wnt proteins are activated during cardiac repair and the wound healing process.^[^ ^7^ ^]^ Moreover, inhibition of Wnt/β‐catenin signaling results in the inability of neonatal cardiomyocytes to proliferate.^[^ ^8^ ^]^ These findings indicate that Wnt/β‐catenin signaling regulates cardiomyocyte proliferation and cardiac repair. Therefore, targeting the Wnt/β‐catenin signaling pathway holds promise for treating myocardial damage. The Krüppel‐like factor (KLF) family is a group of zinc finger transcription factors that play critical roles in various cardiovascular biological processes, including development, differentiation, proliferation, and apoptosis.^[^ ^9^, ^10^, ^11^ ^]^ Several KLF family members have been identified as key regulators of heart development and cardiomyocyte proliferation and are related to heart function under both pathological and physiological conditions. In response to hemodynamic alterations, KLF2 promotes cardiac regeneration by activating the Notch signaling pathway.^[^ ^12^ ^]^ Transcriptional reprogramming‐mediated alterations in KLF4 expression can reverse the transformation of the adult heart from a nonregenerative state back to the renewable fetal state and promote myocardial recovery.^[^ ^13^ ^]^ Loss of a *Klf13* allele significantly increases the penetrance of cardiac abnormalities during heart development in mice.^[^ ^14^ ^]^ In addition, inhibition of KLF15 facilitates cardiomyocyte dedifferentiation and proliferation in the damaged heart.^[^ ^15^ ^]^ Interestingly, the multifaceted function of KLF depends on Wnt/β‐catenin signaling pathway activation. For example, KLF15 regulates Wnt signaling to control homeostasis and disease in the adult heart.^[^ ^16^ ^]^ KLF3 promotes the growth and metastasis of gastric cancer via activation of the Wnt/β‐catenin signaling pathway.^[^ ^17^ ^]^ KLF1 is one of the main members of the KLF family, and much focus has been placed on its role in erythropoiesis and the regulation of mitochondrial function.^[^ ^18^, ^19^ ^]^ A recent study revealed that KLF1 is a crucial cardiomyogenic trigger in zebrafish.^[^ ^20^ ^]^ KLF1 also plays an important role of participating in the regulation of Wnt/β‐catenin pathway.^[^ ^21^, ^22^ ^]^ Nevertheless, little is known about the role of KLF1 in mammalian cardiomyocyte proliferation and cardiac regeneration and whether KLF1 regulates cardiac regeneration through the Wnt/β‐catenin signaling pathway.

In this study, we demonstrated that KLF1 promotes cardiomyocyte proliferation and cardiac regeneration in adult mice. Using cell and animal models, we found that KLF1 is essential for cardiomyocyte proliferation and cardiac regeneration in neonatal mice. In addition, we verified that forced KLF1 overexpression increases cardiomyocyte proliferation and cardiac regeneration in adult mice following MI. Through RNA‐seq and ATAC‐seq analyses, we revealed that KLF1 induces cardiomyocyte regeneration via transcriptional and epigenetic reprogramming. Furthermore, we discovered that KLF1 promotes cardiomyocyte proliferation via the Wnt/β‐catenin signaling pathway. In conclusion, our findings highlight the role of the KLF1/Wnt/β‐catenin signaling pathway in cardiomyocyte proliferation and cardiac regeneration and suggest that the modulation of this pathway may be a potential therapeutic strategy for heart failure.

## 2 Results

### 2.1 KLF1 Facilitates Cardiomyocyte Proliferation In Vivo and In Vitro

**Figure 1:**
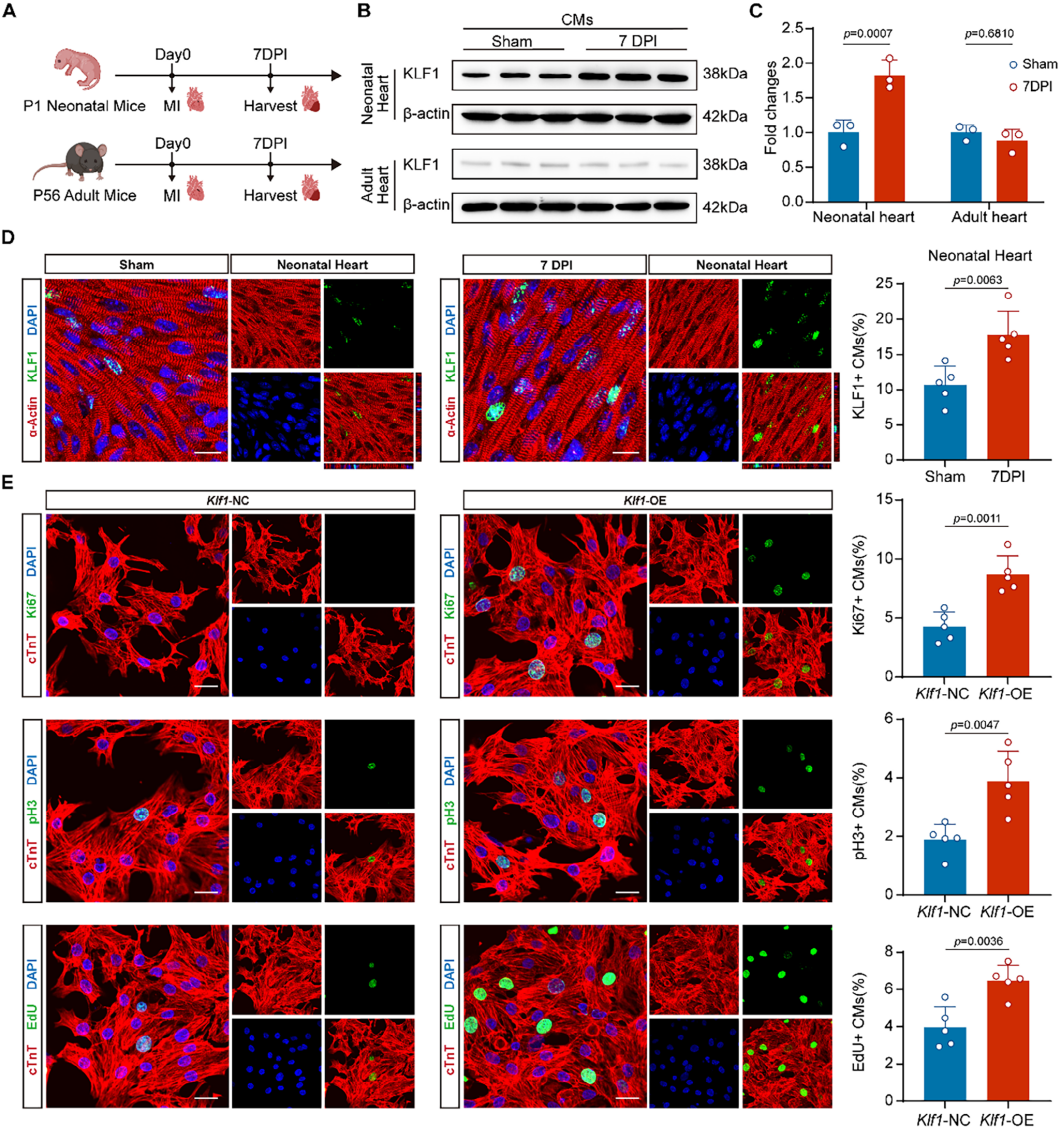
KLF1 facilitates cardiomyocyte proliferation in vivo and in vitro. A) Schematic of the experimental design: MI was induced in P1 neonatal mice and P56 adult mice, and the hearts were harvested at 7 DPI for subsequent experiments. B) The protein levels of KLF1 in isolated neonatal and adult cardiomyocytes at 7 DPI were measured via Western blot. C) Bar graph shows the quantification of KLF1 protein levels in isolated neonatal and adult cardiomyocytes in Figure 1B (n = 3; two‐way ANOVA). D) Representative confocal microscopy images of heart sections from neonatal mice at 7 DPI (a‐Actin, red; KLF1, green; and DAPI, blue). Scale bar, 15 µm. The bar graph shows the percentage of KLF1^+^ cardiomyocytes (n = 5; 2‐tailed unpaired Student's t test). E) Representative confocal microscopy images of isolated cardiomyocytes from postnatal day 7 (P7) mice transduced with AAV9‐*Klf1* for 48 h (cTnT, red; Ki‐67, pH3, and EdU, green; and DAPI, blue). Scale bar, 20 µm. The bar graph shows the percentages of Ki‐67^+^, pH3^+^ and EdU^+^ cardiomyocytes (n = 5; 2‐tailed unpaired Student's t test). KLF1, krüppel‐like factor 1; CMs, cardiomyocytes; DAPI, 4′,6‐diamidino‐2‐phenylindole; P1, postnatal day 1; P56, postnatal day 56; MI, myocardial infarction; DPI, days post infarction; pH3, phospho‐histone H3; EdU, 5‐ethynyl‐2′‐deoxyuridine; AAV9, adeno‐associated virus 9.

To investigate the role of KLF1 in cardiomyocyte proliferation, we examined KLF1 expression levels in the mouse heart at various developmental stages. We firstly isolated cardiomyocytes and non‐cardiomyocytes from the whole heart at representative timepoints according to a previously described protocol (Figure S1A, Supporting Information).^[^ ^23^ ^]^ The effective separation of cardiac components was confirmed by PCM1 staining (Figure S1B–D, Supporting Information).^[^ ^24^ ^]^ Through RT‒qPCR, Western blot and immunofluorescence staining, we next evaluated the dynamic changes in KLF1 expression from infancy (postnatal day 1, P1) to adulthood (postnatal day 56, P56) in isolated cardiomyocytes and heart sections (Figure S1E–G, Supporting Information). The expression of KLF1 was highest in the first days after birth but gradually decreased with age, after which it reached the lowest level and was maintained at a low level in adulthood. Notably, KLF1 expression levels decreased most significantly on postnatal day 7 (P7) (Figure S1E–G, Supporting Information), which coincides with the regenerative windows in mice.^[^ ^3^ ^]^ However, KLF1 exhibited minimal expression in non‐cardiomyocytes and showed no significant change during heart development (Figure S1H,I, Supporting Information). To explore the relationship between reductions in KLF1 expression and heart regenerative capacity, we measured the levels of the cardiomyocyte proliferation markers Ki‐67 and phosphorylated histone H3 (pH3) and analyzed whether they were correlated with the KLF1 expression level at various timepoints (Figure S2A–E, Supporting Information). We found that the percentage of Ki‐67^+^ and pH3^+^ cardiomyocytes was positively correlated with KLF1 expression (Figure S2D,E, Supporting Information). These results suggest that KLF1 may play a role in cardiomyocyte proliferation in mice. To examine whether KLF1 is involved in cardiac regeneration following injury in neonatal mice, myocardial infarction (MI) was induced in P1 mice via permanent ligation of the left anterior descending artery (LAD). We subsequently assessed the expression of KLF1 at 7 days post infarction (DPI) in both cardiomyocytes and non‐cardiomyocytes of the infarcted heart (**Figure** **1**A; Figure S3A, Supporting Information).^[^ ^25^ ^]^ KLF1 was expressed mainly in cardiomyocytes rather than non‐cardiomyocytes in neonatal heart at 7DPI (Figure S3B,C, Supporting Information). Furthermore, non‐cardiomyocytes exhibit scarce KLF1 expression levels and remains unchanged in the hearts of neonatal and adult mice at 7 DPI (Figure S3D,E, Supporting Information). On the contrary, Western blot analysis revealed significantly higher KLF1 expression in the cardiomyocytes of neonatal mice at 7 DPI than in those of mice in the sham group (Figure 1B,C), which coincides with the timepoint at which cardiomyocytes exhibit robust proliferation after heart injury.^[^ ^3^ ^]^ However, there was no significant change in KLF1 expression after MI in the cardiomyocytes of adult mice at P56 (Figure 1B,C). Similarly, immunofluorescence staining revealed an increase in the number of KLF1^+^ cardiomyocytes after MI in neonatal mice (Figure 1D). In contrast, few KLF1^+^ cardiomyocytes were observed in the hearts of adult mice after MI (Figure S3F, Supporting Information).

To elucidate the role of KLF1 in cardiomyocyte proliferation, we isolated primary cardiomyocytes at P7, at which point cardiomyocyte exhibited weak proliferation and negligible KLF1 expression (Figure S1F, Supporting Information). An adeno‐associated virus 9 (AAV9) vector, AAV9‐cTnT‐*Klf1* (AAV9‐*Klf1*), which induces the expression of KLF1 under the control of the cardiac troponin T (cTnT) promoter, was transfected into isolated P7 cardiomyocytes to generate KLF1‐overexpressing cardiomyocytes (*Klf1*‐OE).^[^ ^24^ ^]^ After transfection for 48 hours, immunofluorescence staining confirmed that ≈80% of cardiomyocytes were successfully transfected (Figure S4A,B, Supporting Information), RT‒qPCR and Western blot analysis also verified that KLF1 was successfully overexpressed in cardiomyocytes (Figure S4C–E, Supporting Information). To determine whether forced KLF1 overexpression increases cardiomyocyte proliferation in vitro, we examined the expression of the proliferation markers Ki‐67 and pH3 and the cardiomyocyte marker cTnT. KLF1 overexpression increased the number of Ki‐67^+^ and pH3^+^ cardiomyocytes (Figure 1E). We also assessed DNA synthesis in *Klf1*‐OE cardiomyocytes via an EdU incorporation assay.^[^ ^26^ ^]^ Compared with the *Klf1* negative control (*Klf1*‐NC) group, the proportion of EdU^+^ cardiomyocytes in the *Klf1*‐OE group was obviously greater (Figure 1E). In addition, the mean fluorescence intensity of EdU was greater (Figure 1E; Figure S4F, Supporting Information). These results indicate that KLF1 plays an important role in cardiomyocyte proliferation both in vivo and in vitro.

### 2.2 KLF1 Knockout Inhibits Cardiomyocyte Proliferation and Cardiac Regeneration in Neonatal Mice

To further explore the role of KLF1 in cardiomyocyte proliferation and cardiac regeneration in mice, *Myh6*‐CreEsr1 mice were crossed with *Klf1*‐fl/fl mice to generate *Myh6*‐CreEsr1×*Klf1*‐fl/fl mice (Figure S5A,B, Supporting Information).^[^ ^27^ ^]^ As shown in the schematic in Figure S5A (Supporting Information), cardiomyocyte‐specific KLF1 conditional knockout (*Klf1*‐cKO) mice were generated by continuous tamoxifen injection for five days from P0 to P4; control mice were treated with vehicle instead of tamoxifen.^[^ ^28^ ^]^ Cardiomyocyte‐specific KLF1 knockout was verified by examining mRNA and protein expression levels in the hearts of *Klf1*‐cKO mice (Figure S5C–E, Supporting Information). KLF1 expression was also not significantly elevated after MI in *Klf1*‐cKO mice (Figure S5F–H, Supporting Information). *Klf1*‐cKO did not affect the survival rate or cardiac systolic function in adulthood under physiological conditions (Figures S5I,S6A,B, Supporting Information). Compared with control mice, *Klf1*‐cKO mice presented no differences in heart structure, heart weight or heart‐to‐body weight ratio under physiological conditions (Figure S6C,D, Supporting Information). However, WGA staining at P28 revealed that cardiomyocytes were slightly larger in the hearts of *Klf1*‐cKO mice than in those of control mice (Figure S7A,B, Supporting Information). To determine the effects of *Klf1*‐cKO, we assessed cardiomyocyte proliferation in the hearts of *Klf1*‐cKO and control mice via double immunostaining for a‐Actin and the proliferation markers Ki‐67, pH3, or Aurora‐B at P4. There was no significant difference in the proportion of cells double‐positive for a‐Actin and Ki‐67, pH3 or Aurora‐B in the *Klf1*‐cKO mice compared with control mice (Figure S8A,B, Supporting Information). These findings suggest that KLF1 plays a minimal role in postnatal cardiac development under physiological conditions.

**Figure 2:**
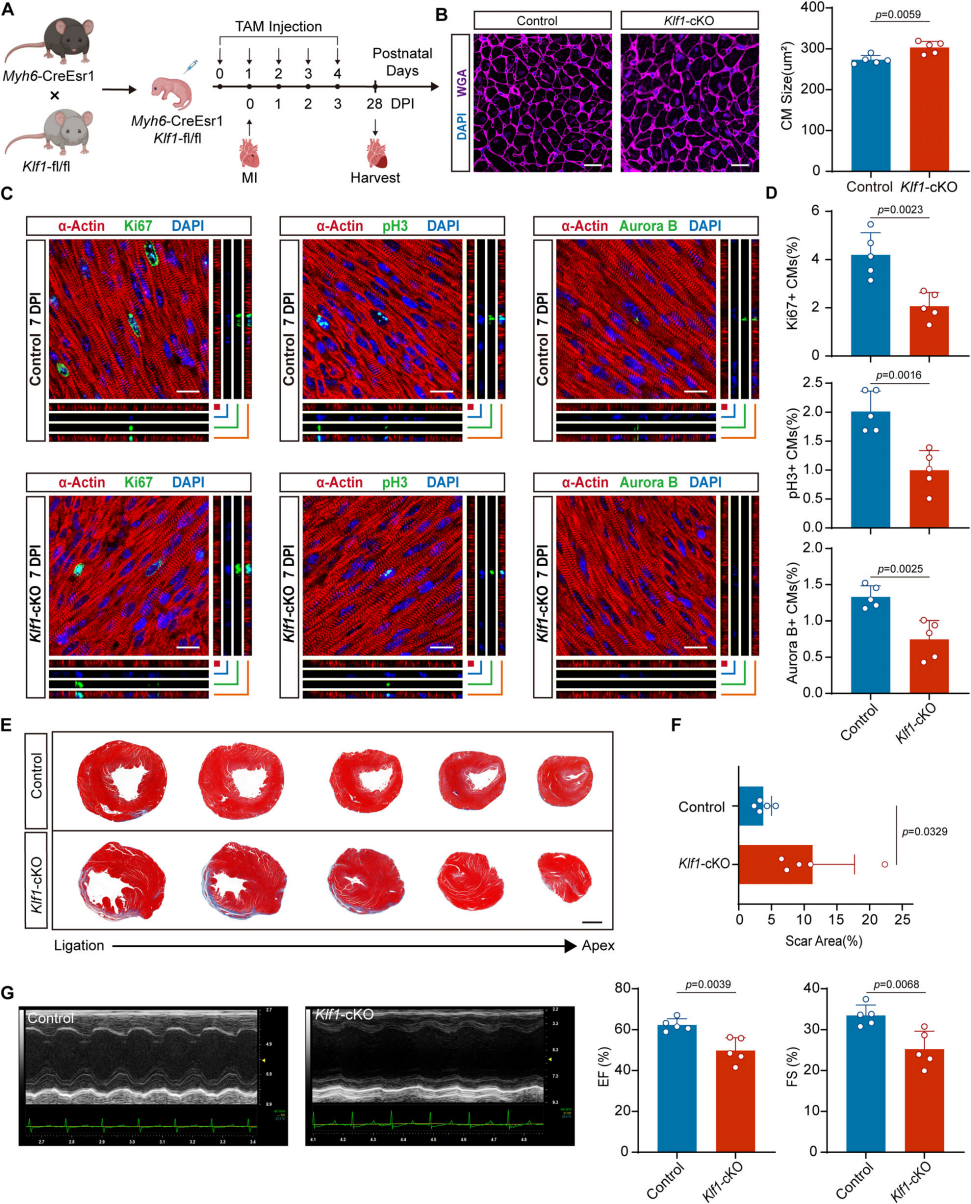
KLF1 knockout inhibits cardiomyocyte proliferation and cardiac regeneration in neonatal mice. A) Schematic of the experimental design: *Klf1*‐cKO mice were generated by crossing *Myh6*‐CreEsr1 mice with *Klf1*‐fl/fl mice, and KLF1 knockout was induced by continuous tamoxifen injection for five days after birth. MI was induced in mice at P1. The hearts were harvested at 7 DPI and 28 DPI for subsequent experiments. B) Representative WGA staining of heart sections from control and *Klf1*‐cKO mice at 28 DPI (WGA, purple; DAPI, blue). Scale bar, 20 µm. Bar graph shows the quantification of average cardiomyocyte size based on WGA staining (n = 5; 2‐tailed unpaired Student's t test). C) Representative confocal microscopy images of heart sections from control and *Klf1*‐cKO mice at 7 DPI (a‐Actin, red; Ki‐67, pH3, and Aurora B, green; and DAPI, blue). Scale bar, 15 µm. D) The bar graph shows the percentages of Ki‐67^+^, pH3^+^, and Aurora B^+^ cardiomyocytes at 7 DPI (n = 5; 2‐tailed unpaired Student's t test). E) Representative Masson's trichrome‐stained cross‐sectional images of control and *Klf1*‐cKO mice cardiac slices sectioned from the ligation site to the apex at 28 DPI. Scale bar, 1mm. F) Bar graph shows the scar areas of heart sections from control and *Klf1*‐cKO mice at 28 DPI (n = 5; 2‐tailed unpaired Student's t test). G) Representative M‐mode echocardiography images of hearts from control and *Klf1*‐cKO mice at 28 DPI. The bar graph shows the EF and FS values for hearts from control and *Klf1*‐cKO mice at 28 DPI (n = 5; 2‐tailed unpaired Student's t test). cKO, conditional knockout; WGA, wheat germ agglutinin; EF, ejection fraction; FS, fraction shortening.

To investigate the role of KLF1 in cardiac regeneration after injury, we constructed a neonatal mouse model of MI.^[^ ^29^ ^]^ Mahmoud, A. I. et al. reported that the hearts of P1 neonatal mice can fully regenerate after MI but gradually lose this ability during postnatal development.^[^ ^30^ ^]^ Accordingly, we induced MI in *Klf1*‐cKO mice at P1. The hearts of the mice were subjected to echocardiography at 28 DPI and then harvested (**Figure** **2**A). Similar to what was observed under physiological conditions, cardiomyocytes were larger in *Klf1*‐cKO mice than in control mice at 28 DPI (Figure 2B). Importantly, Ki‐67, pH3 and Aurora B staining revealed that KLF1 knockout reduced the number of Ki‐67^+^, pH3^+^ and Aurora B^+^ cardiomyocytes at 7 DPI (Figure 2C,D). Moreover, Masson's trichrome staining revealed that *Klf1*‐cKO mice presented more significant postinfarction fibrosis, larger scar areas, and more severe ventricular remodeling than control mice did (Figure 2E,F). These results suggest that KLF1 knockout impairs the ability of cardiomyocyte proliferation and cardiac regeneration in neonatal mice after MI. Additionally, KLF1 knockout severely impaired cardiac function at 28 DPI, as *Klf1*‐cKO mice presented lower fractional shortening (FS) and ejection fraction (EF) values than control mice (Figure 2G). Together, our findings indicate that KLF1 knockout has a minimal effect on cardiac development under physiological conditions but significantly impairs cardiac regeneration and function after MI in neonatal mice. These results indicate that KLF1 is essential for neonatal cardiomyocyte proliferation and cardiac regeneration after heart injury.

### 2.3 Forced KLF1 Overexpression Increases Cardiomyocyte Proliferation and Cardiac Regeneration in Adult Mice after MI

**Figure 3:**
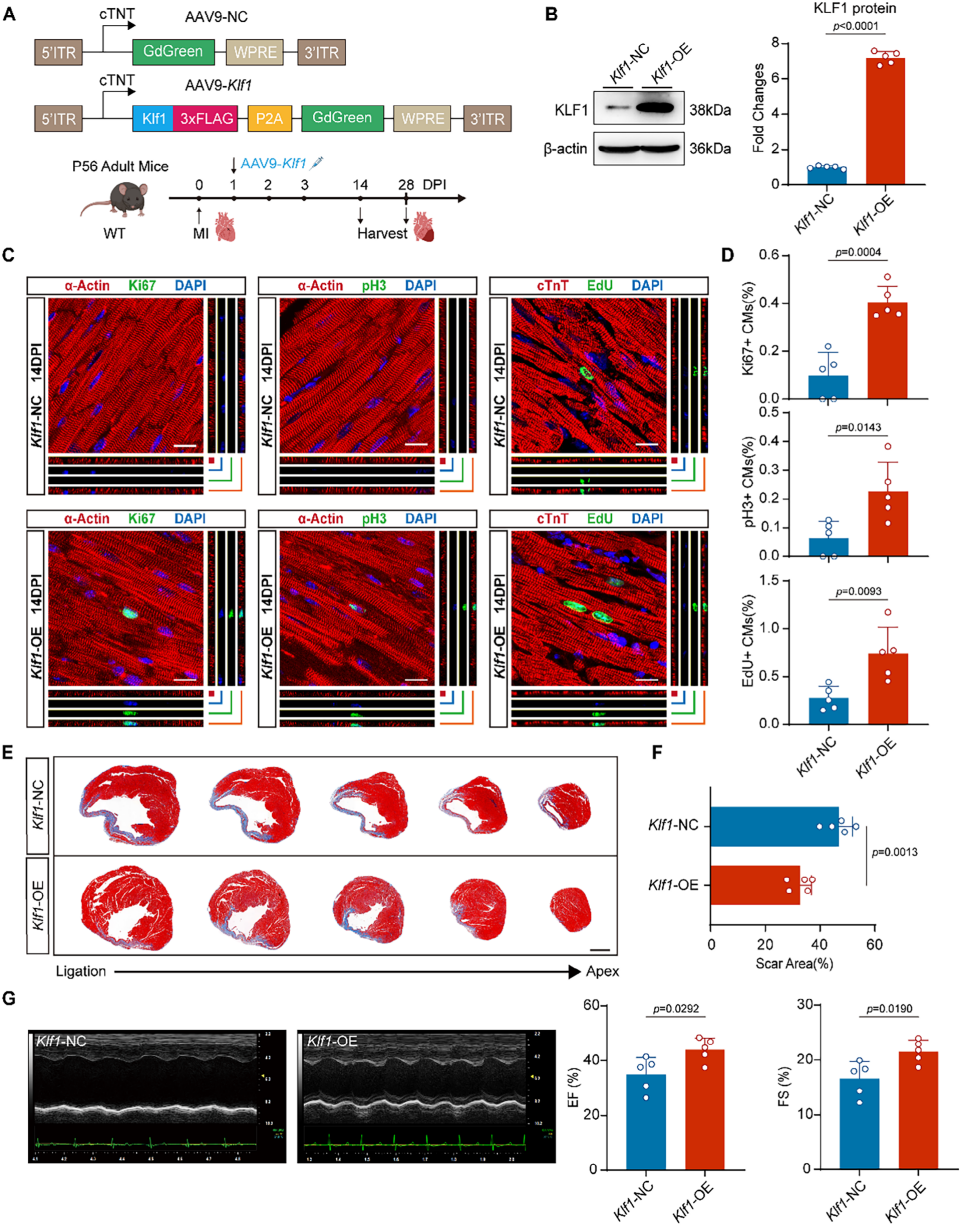
Forced KLF1 overexpression increases cardiomyocyte proliferation and cardiac regeneration in adult mice after MI. A) Schematic of the experimental design: AAV9‐NC or AAV9‐*Klf1*, containing AAV inverted terminal repeats (ITRs), the cardiac TNNT2 (cTnT) promoter, a self‐cleaving 2A peptide (P2A), a woodchuck hepatitis virus posttranscriptional regulatory element (WPRE), and 3FLAG‐*Klf1* and GdGreen, were generated. Eight‐week‐old C57BL/6J mice were treated with AAV9‐NC (*Klf1*‐NC) or AAV9‐*Klf1* (*Klf1*‐OE) via the tail vein on the first day after MI. The hearts were subsequently harvested at 14 DPI and 28 DPI for subsequent experiments. B) The protein levels of KLF1 in the hearts of *Klf1*‐NC and *Klf1*‐OE mice at 14 DPI were measured via Western blot, the bar graph shows the quantification of KLF1 protein levels (n = 5; 2‐tailed unpaired Student's t test). C) Representative confocal microscopy images of heart sections from *Klf1*‐OE mice at 14 DPI (a‐Actin and cTnT, red; Ki‐67, pH3, and EdU, green; DAPI, blue). Scale bar, 15 µm. D) The bar graph shows the percentage of Ki‐67^+^, pH3^+^, and EdU^+^ cardiomyocytes in hearts from *Klf1*‐NC and *Klf1*‐OE mice at 14 DPI (n = 5; 2‐tailed unpaired Student's t test). E) Representative Masson trichrome‐stained cross‐sectional images of *Klf1*‐NC and *Klf1*‐OE mice cardiac slices sectioned from the ligation site to the apex at 28 DPI. Scale bar, 1mm. F) The bar graph shows the scar area of heart sections from *Klf1*‐NC and *Klf1*‐OE mice at 28 DPI (n = 5; 2‐tailed unpaired Student's t test). G) Representative M‐mode echocardiography images of hearts from *Klf1*‐NC and *Klf1*‐OE mice at 28 DPI. The bar graph shows the EF and FS values for hearts from *Klf1*‐NC and *Klf1*‐OE mice at 28 DPI (n = 5; 2‐tailed unpaired Student's t test). WT, wild type; NC, negative control; RT‐qPCR, quantitative real‐time polymerase chain reaction.

Our previous results indicated that KLF1 promotes cardiomyocyte proliferation in vitro. However, KLF1 expression was minimal and did not increase significantly after cardiac injury in adult mice (Figure 1B,C). To evaluate the potential of KLF1 as a therapeutic target for cardiac injury in adult mice, we generated an AAV9 vector loaded with the *Klf1* gene for KLF1 overexpression (*Klf1*‐OE) and a negative control sequence (*Klf1*‐NC). Transgene expression was driven by the cTnT promoter to achieve cardiomyocyte‐specific gene delivery (**Figure** **3**A). MI was induced in adult wild‐type mice on day 0, and the virus was then administered via tail vein injection at 1 DPI (Figure 3A). Immunofluorescence staining confirmed efficient transfection of AAV9 in cardiomyocytes at 14DPI (Figure S9A, Supporting Information). Successful KLF1 overexpression in the adult heart was also verified via Western blot and RT‒qPCR at 14 DPI (Figure 3B; Figure S9B, Supporting Information). Cardiomyocytes with a propensity to proliferate exhibit reduced maturity.^[^ ^31^ ^]^ Therefore, we next assessed whether the overexpression of KLF1 in cardiomyocytes affects cardiomyocyte maturation, including nucleation and cell size,^[^ ^32^ ^]^ in adult mice. To examine nucleation, cardiomyocytes were isolated from *Klf1*‐OE mice at 14 DPI and co‐stained for DAPI and a‐Actin. We determined the proportions of mononuclear, binucleated, and multinucleated (3^+^) cardiomyocytes via fluorescence microscopy.^[^ ^31^ ^]^ The proportion of mononucleated cardiomyocytes was greater and the proportion of binucleated cardiomyocytes was lower in the *Klf1*‐OE group than in the control group, and the proportion of multinucleated cardiomyocytes was comparable between the groups (Figure S9C,D, Supporting Information). These findings indicate that there were more *Klf1*‐OE cardiomyocytes than control cardiomyocytes that were able to proliferate and thus held regenerative potential.^[^ ^26^ ^]^ At 28 DPI, WGA staining revealed less cardiomyocyte hypertrophy in the hearts of *Klf1*‐OE mice than in those of control mice (Figure S9E,F, Supporting Information). These observations prompted us to examine whether there was a change of cardiomyocyte proliferation in *Klf1*‐OE mice. To measure the proliferation of cardiomyocytes, hearts from both groups were sectioned and stained for Ki‐67 and pH3 at 14 DPI. We found that the expression of these markers was significantly increased in the cardiomyocytes of *Klf1*‐OE mice (Figure 3C,D). Moreover, EdU co‐staining assay revealed that the number of EdU^+^cTnT^+^ cardiomyocytes was also significantly greater (by nearly threefold) in the hearts of *Klf1*‐OE mice than in those of *Klf1*‐NC mice (Figure 3C,D). These results revealed that mitosis and DNA replication occurred in the hearts of *Klf1*‐OE mice. Furthermore, Masson's trichrome staining revealed a smaller scar area, less fibrosis and less severe ventricular remodeling in the infarct region during in *Klf1*‐OE mice than in control mice at the same timepoint (Figure 3E,F). Additionally, *Klf1*‐OE mice presented increased EF and FS values, as measured via echocardiography, which indicated that compared with *Klf1*‐NC mice, *Klf1*‐OE mice presented significantly improved cardiac function (Figure 3G). These results indicate that KLF1 overexpression is a promising therapeutic strategy for triggering cardiomyocyte proliferation and cardiac regeneration in adult mice after MI.

### 2.4 KLF1 Overexpression Facilitates Cardiomyocyte Proliferation and Cardiac Regeneration via Transcriptional Reprogramming

**Figure 4:**
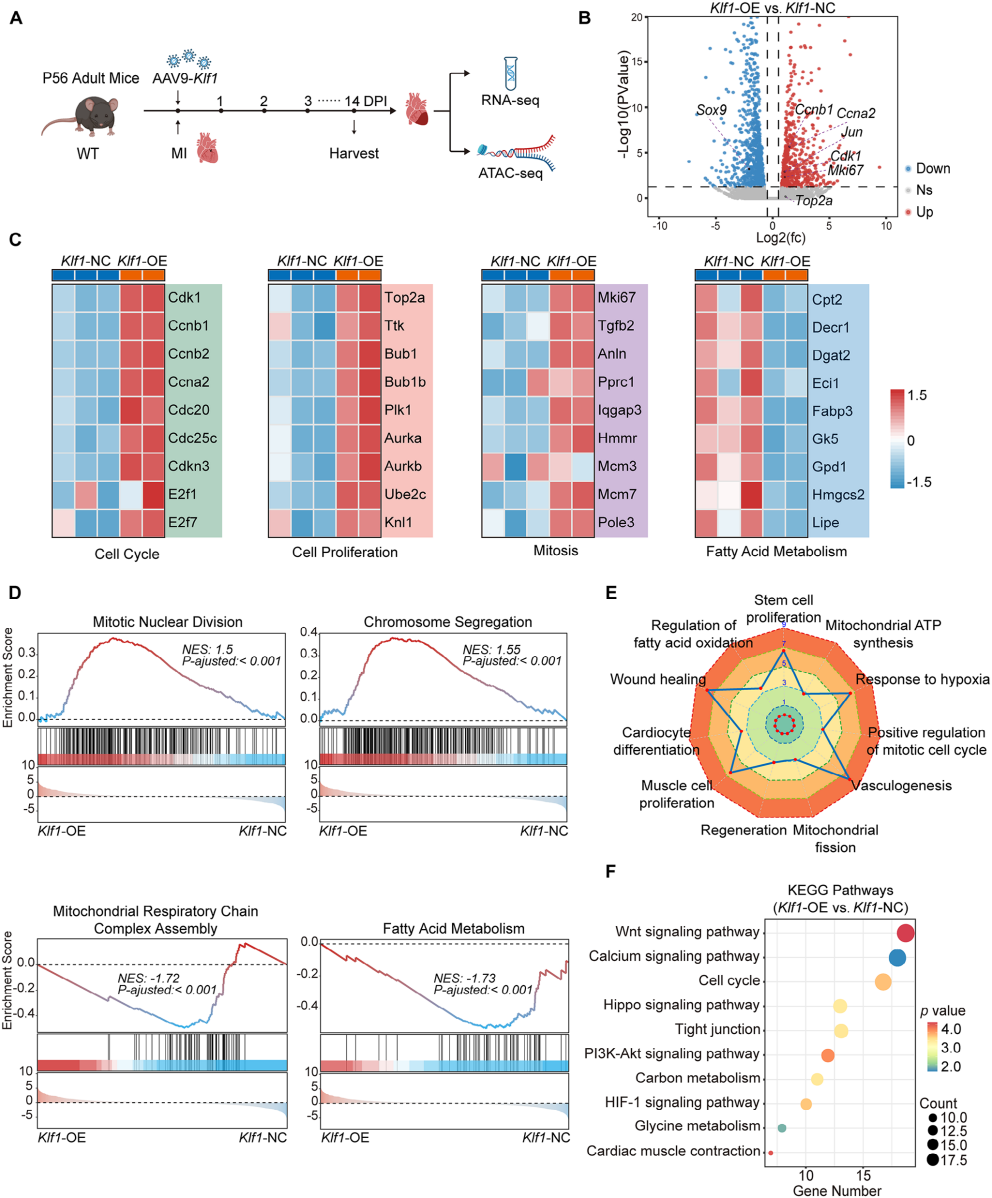
KLF1 overexpression enables cardiomyocyte proliferation and cardiac regeneration by transcriptional reprogramming. A) Schematic of the experimental design for RNA sequencing (RNA‐seq) and ATAC sequencing (ATAC‐seq). Eight‐week‐old C57BL/6J mice were subjected to MI and treated with AAV9‐*Klf1* (*Klf1*‐OE) or AAV9‐NC (*Klf1*‐NC). Heart tissues from *Klf1*‐OE and *Klf1*‐NC mice were subsequently collected at 14 DPI for RNA‐seq and ATAC‐seq analysis. B) Volcano plot of significantly (absolute log2 (fold change) > 1 and *p* adjusted < 0.05) upregulated (red) and downregulated (blue) genes identified by comparing *Klf1*‐OE mice and *Klf1*‐NC mice. C) Heatmaps show the RNA‐seq results of differentially expressed genes related to the cell cycle, cell proliferation, chromosome mitosis and fatty acid metabolism in hearts from *Klf1*‐NC and *Klf1*‐OE mice at 14 DPI. D) Gene set enrichment analysis plot of genes related to mitotic nuclear division, chromosome segregation, mitochondrial respiratory chain complex assembly and fatty acid metabolism that were enriched in *Klf1*‐OE mice. E) Gene Ontology analysis of differentially expressed genes in the hearts of *Klf1*‐NC and *Klf1*‐OE mice. F) Dot plot shows the significant Kyoto Encyclopedia of Genes and Genomes pathways among the differentially expressed proteins identified in *Klf1*‐NC and *Klf1*‐OE mice. Fisher's exact test was used to select significant pathways, which were identified by *p* adjusted < 0.05.

To elucidate the underlying molecular mechanisms by which KLF1 promotes cardiomyocyte proliferation in adult mice, we performed RNA‐seq and ATAC‐seq of the hearts of *Klf1*‐OE and *Klf1*‐NC mice at 14 DPI (**Figure** **4**A). Principal component analysis (PCA) revealed distinct gene expression profiles between the hearts of *Klf1*‐OE mice and those of *Klf1*‐NC mice (Figure S10A, Supporting Information). Further analysis of the RNA‐seq data revealed that 1945 genes were significantly differentially expressed. Specifically, 785 of these genes were upregulated, whereas 1160 were downregulated (Figure 4B). Consistent with the results of the phenotypic examinations, the genes whose expression was upregulated in the hearts of *Klf1*‐OE mice, such as *Ccnb2*, *Top2a*, and *Mki67*, were related to the cell cycle, cell proliferation, and mitosis. Moreover, the expression of markers of fibrosis, such as *Sox9*, was downregulated (Figure 4B,C).^[^ ^33^, ^34^ ^]^ Through gene set enrichment analysis (GSEA), we found that in the hearts of *Klf1*‐OE mice, the differentially expressed genes (DEGs) were enriched mainly in the mitotic nuclear division and chromosome segregation pathways (Figure 4D). Furthermore, the downregulated genes in the hearts of *Klf1*‐OE mice were enriched in the mitochondrial respiratory chain complex assembly pathway (Figure 4D). The mitochondria in the hearts of *Klf1*‐OE mice presented a reduced number of cristae and enlarged matrices (Figure S10B, Supporting Information), which are associated with cardiac regeneration.^[^ ^35^ ^]^ In addition, in the hearts of *Klf1*‐OE mice, the fatty acid metabolism pathway was suppressed, and the transcription of related genes was decreased (Figure 4C,D). Previous studies reported that a metabolic shift in neonatal mouse cardiomyocytes from glycolysis to fatty acid metabolism during postnatal development,^[^ ^36^, ^37^, ^38^ ^]^ and inhibition of fatty acid oxidation could promote cardiac regeneration after ischemia in adult mice.^[^ ^39^ ^]^ Therefore, our results suggest that the metabolic shift induced by the overexpression of KLF1 in cardiomyocytes favors dedifferentiation and regeneration. Gene Ontology (GO) analysis revealed that KLF1 overexpression led to widespread promotion of biological processes related to cardiac regeneration, such as positive regulation of the mitotic cell cycle, stem cell proliferation, mitochondrial fission, angiogenesis and wound healing (Figure 4E).^[^ ^40^ ^]^ Kyoto Encyclopedia of Genes and Genomes (KEGG) analysis also revealed that the DEGs were enriched in many signaling pathways that have been shown to be positively associated with the regulation of cardiomyocyte proliferation and cardiac regeneration, such as the Wnt signaling pathway and the PI3K‐AKT signaling pathway.^[^ ^28^, ^41^, ^42^ ^]^ Among these pathways, the changes in the Wnt signaling pathway were the most significant (Figure 4F). These results indicate that KLF1 overexpression induces cardiomyocyte proliferation via transcriptional reprogramming during the process of cardiac regeneration after MI.

### 2.5 KLF1 Promotes Cardiomyocyte Proliferation and Cardiac Regeneration via the Wnt/β‐Catenin/c‐Myc Signaling Pathway

**Figure 5:**
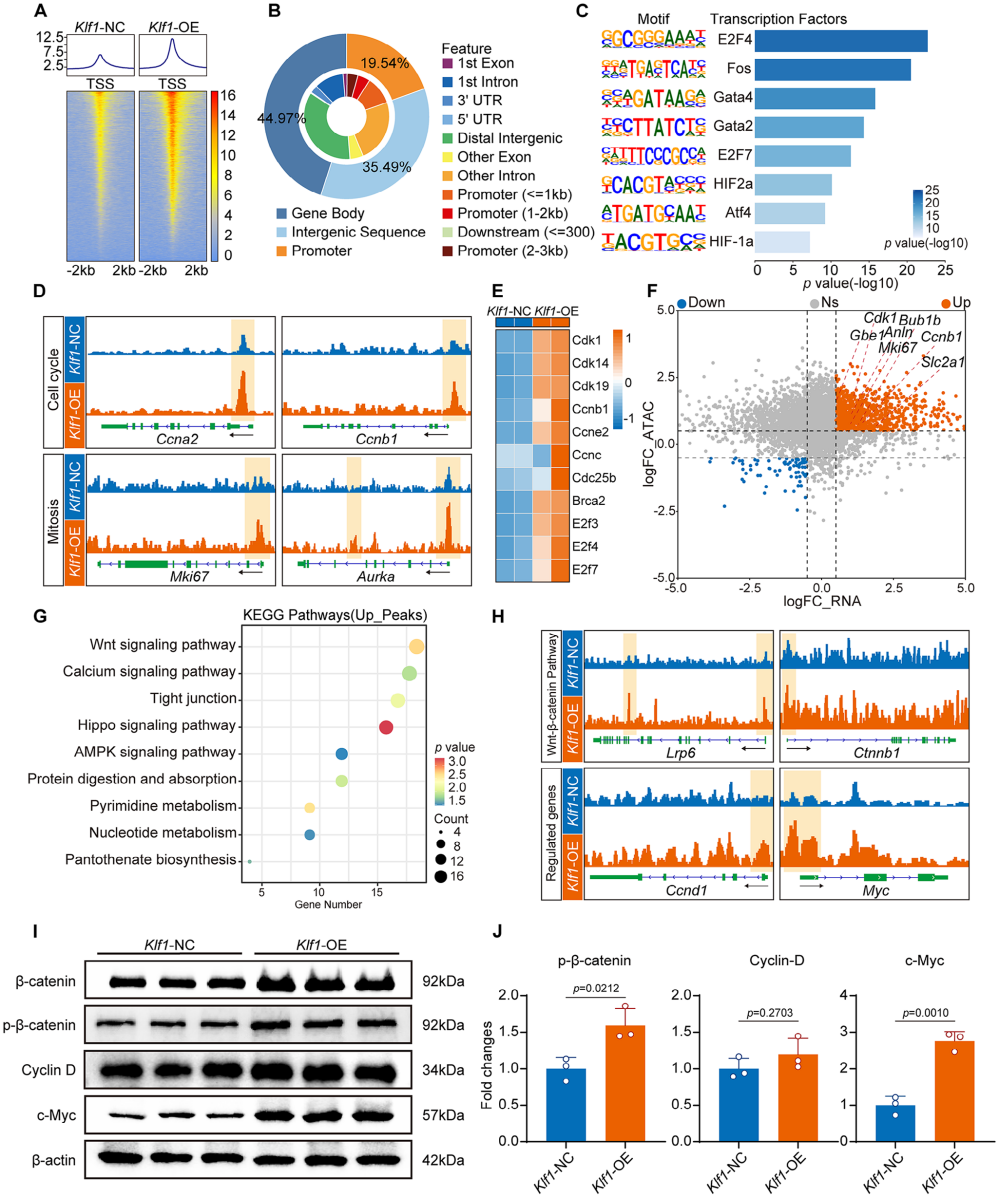
KLF1 promotes cardiomyocyte proliferation and cardiac regeneration via the Wnt/β‐catenin/c‐Myc signaling pathway. A) Heatmaps for differentially enriched ATAC‐seq peaks in hearts from *Klf1*‐NC and *Klf1*‐OE mice at 14 DPI. B) Pie chart shows the genomic distribution of accessible regions in hearts from *Klf1*‐NC and *Klf1*‐OE mice at 14 DPI. C) DNA‐binding motifs enriched in the open chromatin regions of hearts from *Klf1*‐OE mice at 14 DPI. D) Gene tracks show the increased ATAC‐seq peaks of representative cell cycle and mitosis genes (*Ccna2*, *Ccnb1*, *Mki67* and *Aurka*) in hearts from *Klf1*‐OE mice at 14 DPI. E) Heatmaps show the ATAC‐seq analysis of upregulated genes related to the cell cycle in hearts from *Klf1*‐NC and *Klf1*‐OE mice at 14 DPI. F) Quadrantal diagram depicting the overlap of significantly differentially expressed genes from bulk RNA‐seq and genes with significantly variational peaks from ATAC‐seq data. Both upregulated (orange); both downregulated (blue). G) Dot plot shows the significant Kyoto Encyclopedia of Genes and Genomes pathways associated with the overlapping DEGs between the bulk RNA‐seq and ATAC‐seq data. Fisher's exact test was used to select significant pathways, which were identified by *p* adjusted < 0.05. H) Gene tracks show the increased ATAC‐seq peaks of representative genes of the Wnt‐β‐catenin signaling pathway (*Lrp6*, *Ctnnb1*, *Ccnd1* and *Myc*) in the hearts of *Klf1*‐OE mice at 14 DPI. I) Western blot of representative proteins of the Wnt‐β‐catenin pathway in hearts from *Klf1*‐NC and *Klf1*‐OE mice at 14 DPI. J) Bar graph shows the protein levels of representative proteins of the Wnt‐β‐catenin pathway in hearts from *Klf1*‐NC and *Klf1*‐OE mice at 14 DPI (n = 3; 2‐tailed unpaired Student's t test).

Our previous study revealed that KLF1 overexpression significantly activates the Wnt signaling pathway in cardiomyocytes, which is involved in the development, proliferation, and regeneration of cardiomyocytes, both under pathological and physiological conditions.^[^ ^43^, ^44^, ^45^ ^]^ Therefore, to investigate how KLF1 regulates the Wnt signaling pathway to manipulate cardiomyocyte proliferation at the epigenetic level, we performed ATAC‐seq of the hearts of both *Klf1*‐NC and *Klf1*‐OE mice at 14 DPI.^[^ ^46^ ^]^ Analysis of the enrichment of ATAC‐seq peaks at genome‐wide regions surrounding transcription start sites (± 2 kb; TSS) revealed that KLF1 overexpression induced a global increase in chromatin accessibility (**Figure** **5**A). We annotated the differential peaks according to their genomic distribution, and the majority of the peaks were mapped to intronic and intergenic regions (Figure 5B). To link changes in accessible chromatin with specific transcription factors (TFs), HOMER motif analysis of the enriched open chromatin regions was performed. We identified significantly enriched TFs involved in cardiomyocyte dedifferentiation (*Gata4* and *Gata2*),^[^ ^47^, ^48^, ^49^ ^]^ proliferation (*E2f4* and *Fos*)^[^ ^50^, ^51^ ^]^ and cardiac regeneration (*Atf4* and *Hif‐1a*)^[^ ^52^, ^53^ ^]^ in the hearts of *Klf1*‐OE mice (Figure 5C). Moreover, consistent with our RNA‐seq results, a snapshot of the ATAC‐seq profiles revealed more accessible peaks at promoters of known cell cycle (*Ccna2* and *Ccnb1*)‐ and mitosis (*Mki67* and *Aurka*)‐ related genes in the hearts of *Klf1*‐OE mice than in those of *Klf1*‐NC mice (Figure 5D,E). To better understand the mechanism by which KLF1 promotes cardiomyocyte proliferation at the transcriptional level, we combined the ATAC‐seq and RNA‐seq results for subsequent analysis.^[^ ^41^ ^]^ The integrated analysis revealed 681 overlapping DEGs (266 upregulated and 415 downregulated genes) between the ATAC‐seq and RNA‐seq data (Figure 5F). Numerous upregulated genes from the RNA‐seq data that were strongly associated with the cell cycle and cell division presented more accessible promoter regions (Figure 5F). To further elucidate the functions of these overlapping upregulated genes, we conducted KEGG enrichment analysis. Notably, genes enriched in the Wnt signaling pathway were more highly expressed in the hearts of *Klf1*‐OE mice than in those of *Klf1*‐NC mice (Figure 5G). Genes encoding upstream (*Lrp6*), core (*Ctnnb1*) and downstream (*Ccnd1* and *Myc*) factors of the Wnt/β‐catenin pathway presented more accessible promoters and higher gene expression levels in the hearts of *Klf1*‐OE mice than in those of *Klf1*‐NC mice (Figure 5H).^[^ ^7^, ^45^, ^54^ ^]^ For further validation, we performed Western blot analysis of β‐catenin expression and confirmed that the Wnt/β‐catenin pathway was significantly activated in the hearts of *Klf1*‐OE mice (Figure 5I,J). Furthermore, KLF1 overexpression increased the phosphorylation of β‐catenin on serine 675, which is necessary for promoting β‐catenin transcriptional activity and downstream gene expression (Figure 5I,J).^[^ ^55^, ^56^ ^]^ We also assessed the expression of β‐catenin‐regulated target genes (cyclin D and c‐Myc).^[^ ^45^ ^]^ Western blot revealed that c‐Myc expression was significantly increased, whereas no obvious change in cyclin D expression was detected (Figure 5I,J). Our findings suggest that Wnt/β‐catenin signaling is significantly activated in the hearts of *Klf1*‐OE mice and that KLF1 may regulate cardiomyocyte proliferation and cardiac regeneration through Wnt/β‐catenin/c‐Myc signaling.

### 2.6 Inhibition of the Wnt/β‐Catenin Pathway Impairs KLF1‐Mediated Cardiomyocyte Proliferation and Cardiac Regeneration

**Figure 6:**
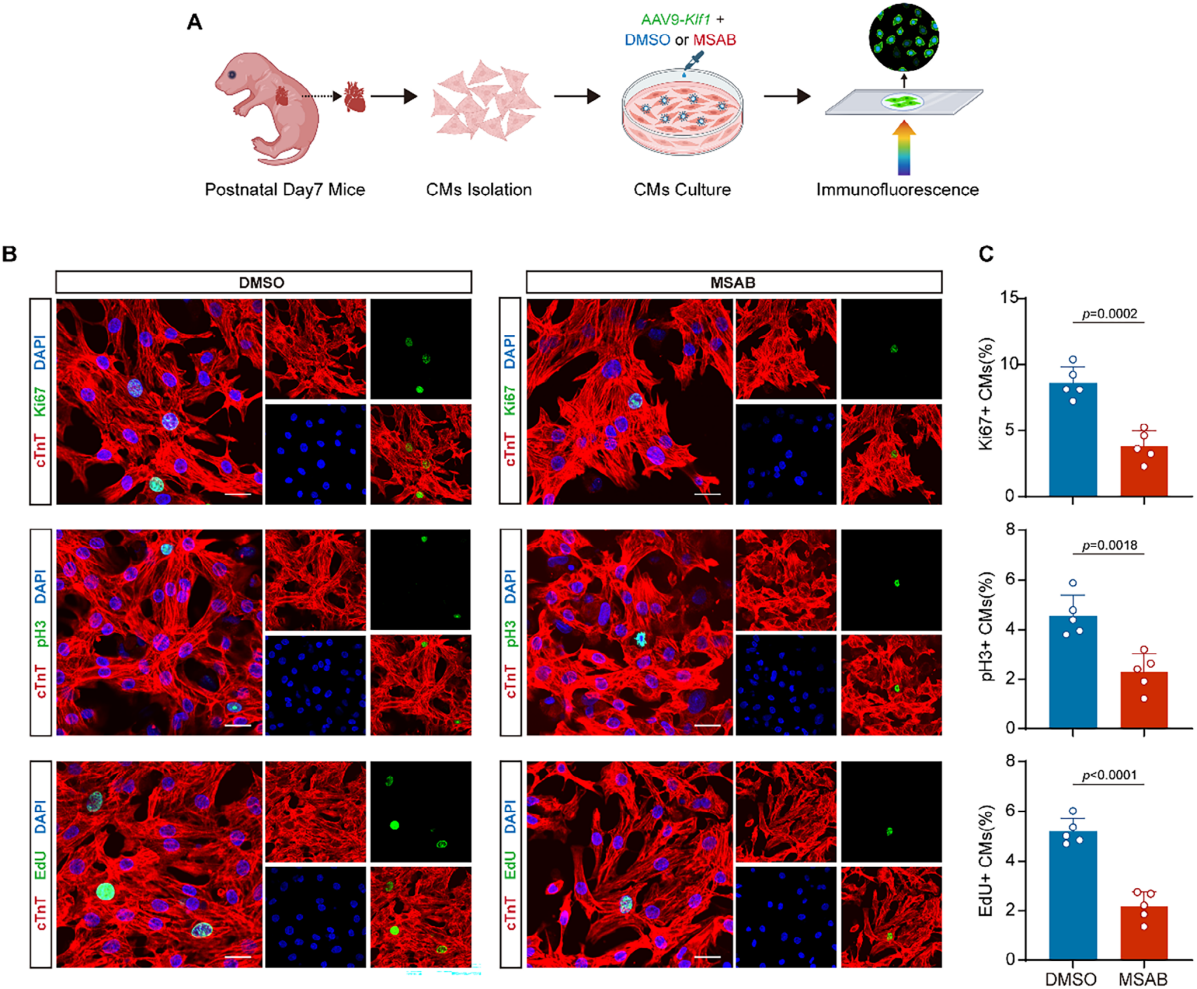
Inhibition of the Wnt‐β‐catenin pathway hampers KLF1 mediated cardiomyocyte proliferation in vitro. A) Schematic of the experimental design to disrupt KLF1 and the Wnt pathway in neonatal mouse cardiomyocytes: cardiomyocytes were isolated and cultured from the hearts of P7 neonatal mice. AAV9‐*Klf1* with MSAB or vehicle were added to the medium for another 48 h of culture. The proliferation of cardiomyocytes was subsequently detected via immunofluorescence. B) Representative confocal microscopy images of isolated P7 mouse cardiomyocytes cultured with AAV9‐*Klf1* and MSAB for 48 h (cTnT, red; Ki‐67, pH3, and EdU, green; and DAPI, blue). Scale bar, 20 µm. C) The bar graph shows the percentages of Ki‐67^+^, pH3^+^ and EdU^+^ cardiomyocytes (n = 5; 2‐tailed unpaired Student's t test).

**Figure 7:**
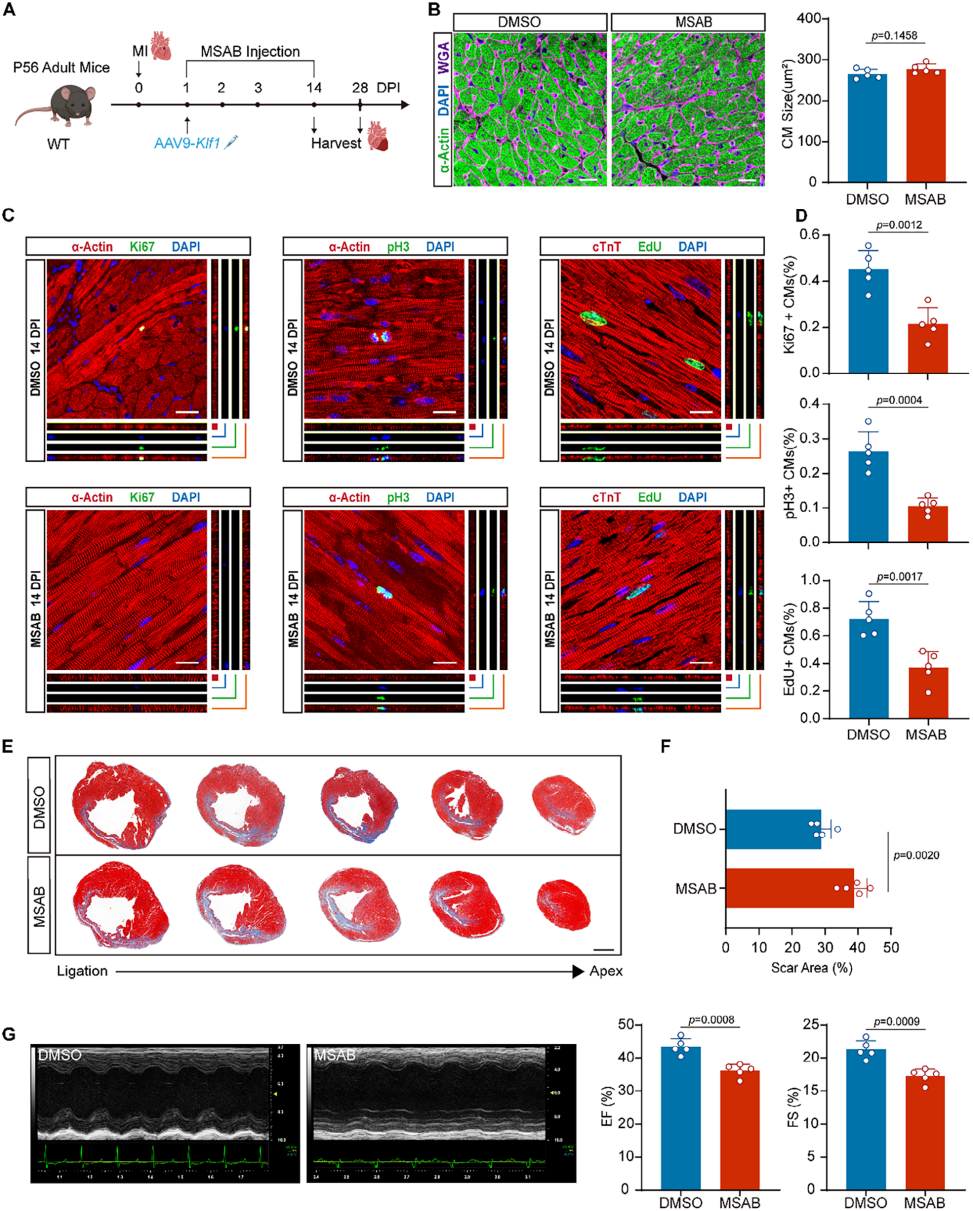
Inhibition of the Wnt/β‐catenin pathway impairs KLF1 mediated cardiomyocyte proliferation and cardiac regeneration after MI. A) Schematic of the experimental design: 8‐week‐old C57BL/6J mice were treated with AAV9‐*Klf1* via the tail vein on the first day after MI. DMSO or MSAB was then administered intraperitoneally every other day. The hearts were harvested at 14 DPI and 28 DPI for analysis. B) Representative WGA staining of heart sections from *Klf1*‐OE mice treated with MSAB at 28 DPI (WGA, purple; a‐Actin, green; and DAPI, blue). Scale bar, 20 µm. Bar graph shows the quantification of average cardiomyocyte size based on WGA staining (n = 5; 2‐tailed unpaired Student's t test). C) Representative confocal microscopy images of heart sections from *Klf1*‐OE mice injected with DMSO or MSAB at 14 DPI (a‐Actin, red; Ki‐67, pH3, and EdU, green; and DAPI, blue). Scale bar, 15 µm. D) Bar graph shows the percentage of Ki‐67^+^, pH3^+^, and EdU ^+^ cardiomyocytes in hearts from *Klf1*‐OE mice treated with DMSO or MSAB at 14 DPI (n = 5; 2‐tailed unpaired Student's t test). E) Representative Masson trichrome‐stained cross‐sectional images of *Klf1*‐OE mice hearts treated with DMSO or MSAB sectioned from the ligation site to the apex at 28 DPI. Scale bar, 1mm. F) Bar graph shows the scar area of heart sections from *Klf1*‐OE mice treated with DMSO or MSAB at 28 DPI (n = 5; 2‐tailed unpaired Student's t test). G) Representative M‐mode echocardiography of hearts from *Klf1*‐OE mice treated with DMSO or MSAB at 28 DPI. The bar graph shows the EF and FS values for hearts from *Klf1*‐OE mice treated with DMSO or MSAB at 28 DPI (n = 5; 2‐tailed unpaired Student's t test).

**Figure 8:**
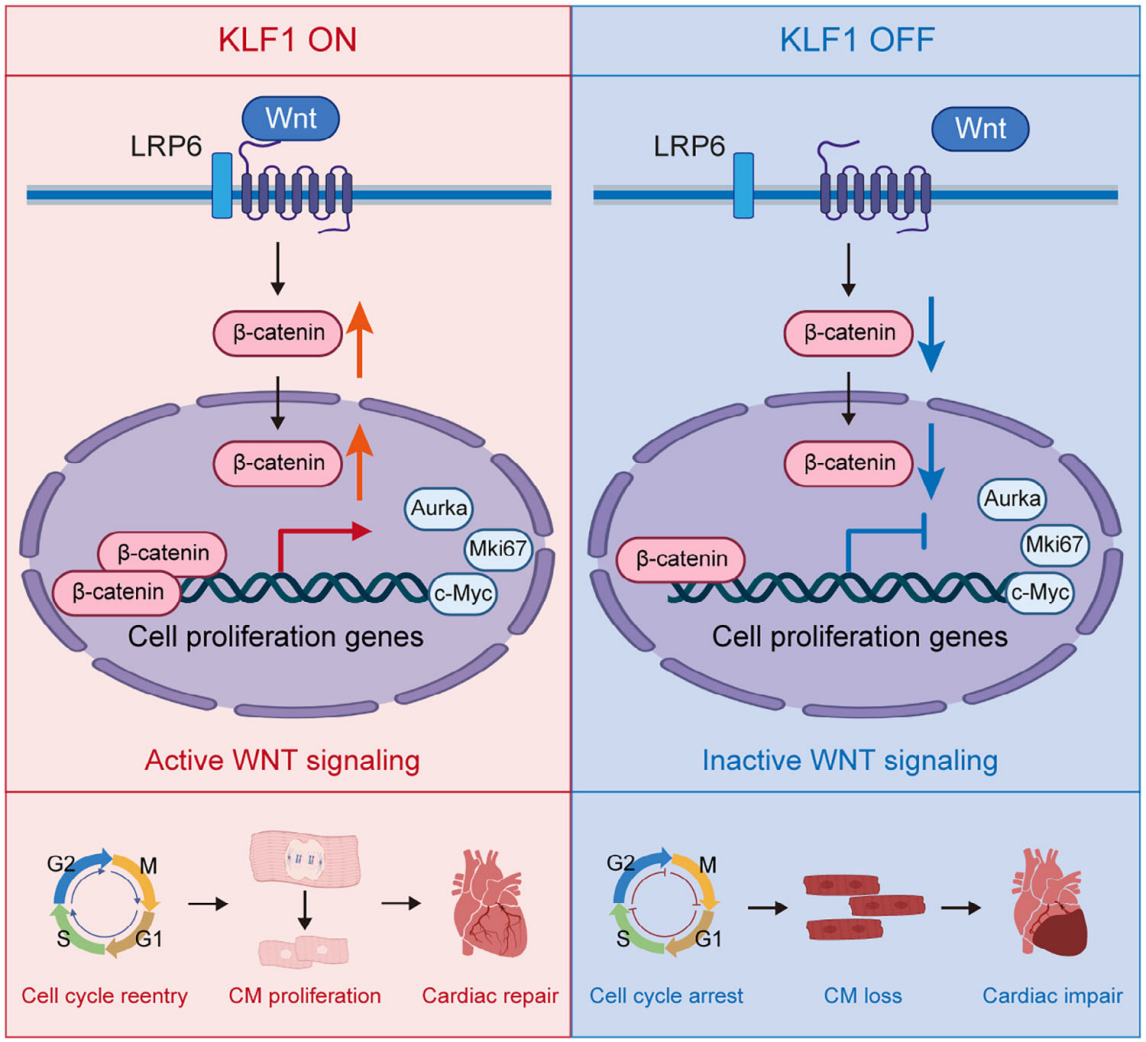
KLF1 Promotes Cardiomyocyte Proliferation and Heart Regeneration through Regulation of Wnt/β‐catenin signaling Pathway. In KLF1‐on (neonatal and *Klf1* overexpression hearts) models, KLF1 regulates Wnt/β‐catenin signaling pathway and the expression of cell proliferation‐related genes via transcriptional and epigenetic mechanisms, which induces cardiomyocyte proliferation and cardiac regeneration in mice after myocardial infarction. However, KLF1‐off (adult and *Klf1* knockout hearts) models inhibit the active of Wnt/β‐catenin signaling and cell proliferation‐related genes expression, which results in cardiomyocyte loss and cardiac impair.

To investigate the role of the Wnt/β‐catenin pathway in KLF1‐mediated cardiomyocyte proliferation, we treated isolated cardiomyocytes with MSAB, a potent and selective inhibitor of β‐catenin, at a concentration of 5 µM.^[^ ^42^ ^]^ All the isolated cardiomyocytes were cultured with AAV9‐*Klf1* and MSAB separately or together for 48 h. Subsequently, immunofluorescence staining was performed to assess Ki‐67 and pH3 expression and EdU incorporation in cardiomyocytes (**Figure** **6**A). As expected, MSAB decreased the proportion of Ki‐67^+^, pH3^+^ and EdU^+^ cardiomyocytes with AAV9‐*Klf1* treatment (Figure 6B,C). These results suggest that KLF1 overexpression promotes cardiomyocyte proliferation through Wnt/β‐catenin signaling. To verify the effect of blocking the Wnt/β‐catenin pathway in vivo, we treated *Klf1*‐OE mice with MSAB or DMSO. After MI, adult mice were treated with AAV9‐*Klf1* at 1 DPI. The mice were subsequently injected intraperitoneally with MSAB or DMSO once every other day for 14 days after infarction (**Figure** **7**A). Although there was no significant difference in cardiomyocyte size (Figure 7B), the numbers of Ki‐67^+^, pH3^+^ and EdU^+^ cardiomyocytes were decreased in the hearts of *Klf1*‐OE mice after treatment with MSAB (Figure 7C,D), which suggests that the ability of KLF1 to promote cardiomyocyte proliferation was attenuated by blocking Wnt/β‐catenin signaling. Masson's trichrome staining revealed that the inhibition of Wnt/β‐catenin signaling was sufficient to exacerbate fibrotic scarring in the hearts of *Klf1*‐OE mice after MI (Figure 7E,F). As expected, compared with vehicle treatment, MSAB treatment resulted in worse EF and FS values, indicating impaired cardiac function in the hearts of *Klf1*‐OE mice (Figure 7G). Taken together, these results suggest that KLF1 overexpression promotes cardiomyocyte proliferation and cardiac regeneration through the Wnt/β‐catenin signaling pathway (**Figure** **8**).

## 3 Discussion

Following intensive and extensive research in recent years, the molecular mechanisms underlying the loss of proliferative capacity in cardiomyocytes during the postnatal stages have been profoundly understood.^[^ ^5^, ^57^ ^]^ However, despite the rapid advancements in fundamental research, efficient translational methods to promote cardiomyocyte proliferation and cardiac regeneration in adult mammals have yet to be developed based on these meaningful findings.^[^ ^58^ ^]^ In our study, we identified a novel pathway regulating cardiomyocyte proliferation involving the zinc finger transcription factor KLF1. Specifically, KLF1 expression markedly decreased during the early postnatal period and remained at low levels at later time points, coinciding with the time points at which the myocardium‐regenerative capacity is lost in mice. By altering KLF1 expression in cardiomyocytes in an animal model, we demonstrated that KLF1 promotes cardiomyocyte proliferation and cardiac regeneration. Mechanistic investigations in vitro and in vivo revealed that the effects of KLF1 on cardiomyocytes are dependent on the Wnt/β‐catenin signaling pathway. These studies reveal a novel mechanism underlying the role of the KLF1/Wnt/β‐catenin signaling pathway in cardiomyocyte proliferation and cardiac regeneration in mice, providing a potential therapeutic target for patients with myocardial damage and heart failure.

In this study, KLF1 expression markedly decreased during the early postnatal period, and trace expression maintained at different time points throughout adulthood, coinciding with the time points at which the myocardium‐regenerative capacity is lost in mice.^[^ ^3^ ^]^ For further investigation, we generated cardiomyocyte‐specific KLF1 knockout mice. *Klf1* knockout did not affect cardiac development or cardiomyocyte proliferation under physiological conditions. However, *Klf1* knockout severely decreased the proliferation of cardiomyocytes during the period of cardiac regeneration and led to incomplete cardiac regeneration and deteriorated cardiac function in mice that underwent myocardial infarction at P1. This change may have been due to the low expression of KLF1 in cardiomyocytes during normal postnatal development but the significant upregulation of KLF1 expression following myocardial infarction in neonatal mice. Furthermore, given the widespread application prospects of adeno‐associated virus (AAV)‐mediated gene therapy in the study of heart disease,^[^ ^59^ ^]^ we overexpressed KLF1 in cardiomyocytes. By applying an AAV9‐mediated therapeutic strategy, we successfully induced cardiomyocyte proliferation both in vitro and in vivo, as well as cardiac regeneration and myocardial recovery in adult mice after MI. Taken together, these results suggest that KLF1 plays a critical role in cardiomyocyte proliferation and that increasing KLF1 expression is an effective means to improve cardiac function after MI.

Cardiomyocyte proliferation and cardiac regeneration are complex and multifaceted processes. Cardiomyocytes need to undergo mitosis and form complete functional complexes through multiple biological processes, including both endogenous and exogenous processes such as cell cycle reentry, metabolic conversion, mitochondrial function, and intracellular signal transduction.^[^ ^52^, ^60^, ^61^, ^62^ ^]^ Moreover, cardiomyocyte proliferation and cardiac regeneration are regulated by multiple factors at the transcriptome and epigenetic levels. Previous studies have demonstrated that KLF1 can increase cellular proliferation, orchestrate metabolic processes, and modulate mitochondrial function in many ways.^[^ ^19^, ^63^ ^]^ By performing RNA‐seq analysis, we revealed that KLF1 overexpression altered the expression of genes associated with the cell cycle, cell proliferation, mitosis, and fatty acid metabolism, which coordinate the proliferation of cardiomyocytes. Moreover, functional enrichment analysis revealed that mitotic nuclear division, chromosome segregation, mitochondrial respiratory chain complex assembly and the fatty acid metabolism signaling pathway, became conducive to cardiomyocyte proliferation after the overexpression of KLF1.^[^ ^39^, ^41^ ^]^ These findings indicate that KLF1 supports the complex biological functions of cardiomyocytes to promote heart regeneration and cardiac repair. By combining RNA‐seq with ATAC‐seq analysis, we found that KLF1 activates various biological processes and signaling pathways at both the epigenetic and transcription levels. The Wnt, Hippo and AMPK signaling pathways, which are indispensable for cardiomyocyte proliferation and cardiac regeneration,^[^ ^6^, ^64^, ^65^ ^]^ were highly activated in the hearts of *Klf1*‐OE mice. Among the multiple altered signal transduction pathways, the Wnt signaling pathway exhibited the most significant changes after KLF1 overexpression. Owing to its elaborate and critical role in the development of the cardiovascular system and heart disease, the Wnt/β‐catenin pathway has been extensively studied. LRP6, which is upstream of Wnt/β‐catenin signaling, drives improvements in cardiac function after MI.^[^ ^54^ ^]^ In the heart, overexpression of β‐catenin, a core component of the Wnt/β‐catenin pathway, strongly promotes the cardiac developmental program and cytoskeletal remodeling, and shares transcription factor‐7 like 2 (Tcf7L2) cooccupying genomic regions with Nkx2.5 and Gata4.^[^ ^66^ ^]^ As essential effectors of the Wnt/β‐catenin pathway, Cyclin D and c‐Myc also directly and positively regulate the cell cycle and cardiac regeneration.^[^ ^45^ ^]^ The upregulation of KLF1 expression can accelerate disease progression by promoting cell proliferation and cancer migration through Wnt/β‐catenin pathway activation.^[^ ^21^, ^67^ ^]^ In our study, we observed that KLF1 overexpression upregulated the expression of all these factors involved in the Wnt/β‐catenin pathway, including the upstream promoter LRP6 and the effector c‐Myc. Experimental blockade of the Wnt/β‐catenin pathway with the selective inhibitor MSAB almost completely abolished KLF1‐mediated cardiomyocyte proliferation and cardiac regeneration, which underscores the importance of the Wnt/β‐catenin pathway for KLF1 function. Taken together, these results suggest that KLF1 overexpression promotes cardiomyocyte proliferation and that cardiac regeneration is dependent on Wnt/β‐catenin signaling.

In summary, the results of this study demonstrate that reduced expression of KLF1 during postnatal development is associated with the loss of myocardial regenerative capacity in mice. KLF1 overexpression is adequate for stimulating cardiomyocyte proliferation and cardiac regeneration in adulthood through the activation of the Wnt/β‐catenin pathway. Our findings highlight the role of KLF1 in cardiomyocyte proliferation and cardiac regeneration and provide new insights for the treatment of MI and heart failure.

## 4 Experimental Section

### Animals

The wild‐type (WT) mice used in the experiment were purchased from Vital River Laboratory Animal Technology Co., Ltd. (Beijing, China). *Klf1‐*fl/fl mice and *Myh6*‐CreEsr1 mice purchased from Model Organisms Center, Inc. (Shanghai, China). The tails of mice were collected for genotype identification via RT‐qPCR and agarose gel electrophoresis. The genotyping primers were listed in Table S1 (Supporting Information). Tamoxifen was injected intraperitoneally at a dose of 100 mg kg^−1^ to induce specific myocardial knockout of the target gene. All the mice used in this study were male, except where otherwise indicated. All experimental mice were housed in an SPF facility at Tongji Medical College of Huazhong University of Science and Technology. All animal experimental procedures were approved by the Experimental Animal Ethics Committee of Huazhong University of Science and Technology (IACUC number 3154).

### Myocardial Infarction in Neonatal Mice

Myocardial infarction in neonatal P1 mice was performed as previously reported.^[^ ^68^ ^]^ In brief, P1 mice were anesthetized at low temperature on ice for 3–4 min. After complete anesthesia, the mice were fixed with tape. Then, with the assistance of a stereomicroscope, the skin and muscles attached to the chest were bluntly separated along the fourth intercostal space. Appropriate pressure was applied to expose the heart, and then, the LAD was ligated with 8‐0 braided silk thread to induce myocardial ischemia in the left ventricle. Subsequently, 6‐0 sutures were used to close the chest cavity. Postoperative mice were placed in a heating blanket for rewarming. In the sham‐operated groups, the same procedures described above were performed without suturing the LAD. At different time points, the hearts were collected for histological and immunohistochemical staining.

### Myocardial Infarction in Adult Mice

Myocardial infarction in adult mice was performed as previously reported.^[^ ^69^ ^]^ Briefly, the mice were anesthetized via intraperitoneal injection of pentobarbital sodium (Solarbio, Beijing, China) at a dose of 40 mg kg^−1^, and their limbs were immobilized following cessation of the toe pressure reflex. After disinfection with alcohol, the hair on the chest of each mouse was shaved. A suture attached to the front upper incisors was pulled taut to allow tracheal intubation. A 20‐G catheter was inserted into the trachea. The catheter was subsequently connected to a mouse ventilator via a Y‐shaped connector. For a 25–30 g mouse, ventilation was performed with a rodent ventilator at a tidal volume of 200–300 µL and a respiratory frequency of 100 breaths per minute as previously reported. After stable breathing, the skin and muscle were bluntly separated, and a small incision was made between the third and fourth intercostal spaces in the chest. The chest was gently squeezed, and the pericardium was removed to expose the heart. Then, the LAD was ligated with 8‐0 threads ≈2 mm below the left atrium to induce myocardial infarction. After ligation, the chest and skin incisions were closed with a 5‐0 nonabsorbable suture, and the postoperative mice were transferred to a heating blanket for rewarming until recovery. In the sham‐operated groups, the same procedures described above were performed without suturing the LAD.

### Isolation of Neonatal Cardiomyocytes

The cardiomyocytes of neonatal mice were isolated in accordance with a previously described protocol with some modifications.^[^ ^24^ ^]^ Briefly, the mouse ventricles were separated and minced into 1–2 mm cubes with scissors and then dissociated in calcium‐ and magnesium‐free HBSS containing 0.25% trypsin and 1 mg mL^−1^ collagenase B with constant stirring. Digestion was performed for ten minutes at room temperature and repeated for 5–7 cycles. The supernatant was collected with an equal volume of DMEM/F12 (Gibco) supplemented with 10% fetal bovine serum (FBS, Gibco) after every digestion step. When the heart tissue was completely digested, the collected supernatant was filtered with a 100 µm diameter cell strainer and centrifuged at 2500 rpm for 8 min to pellet the cells. The cell pellet was resuspended in DMEM/F12 supplemented with 10% FBS at 37 °C and 5% CO2 for 2 h to remove fibroblasts, and cardiomyocytes in the supernatant were plated at an appropriate density on petri dishes precoated with 1% gelatin (Beyotime) for subsequent experiments.

### Isolation of Adult Cardiomyocytes

A simplified Langendorff‐free perfusion method for the isolation of adult cardiomyocytes was performed as previously described.^[^ ^25^ ^]^ Briefly, the chest cavity of an anesthetized mouse was opened to fully expose the heart. The descending aorta and inferior vena cava were severed, and 7 mL of EDTA buffer was immediately injected into the right ventricle to flush the heart. The ascending aorta was clamped with Reynolds forceps, and the heart was removed by cutting behind the clamp. The clamped heart was transferred to a 60‐mm dish containing fresh EDTA buffer. To digest the heart, 10 mL of EDTA buffer, 3 mL of perfusion buffer, and 30 to 50 mL of collagenase buffer were slowly injected sequentially into the left ventricle. The collagenase buffer injection was stopped when the heart was sufficiently digested, and the clamp was removed. The remaining tissue was pulled into 1 mm^3^ sections and dissociated by gentle pipetting. Five milliliters of stop buffer was added to terminate the enzyme reaction, and the isolated cell suspension was passed through a 100 mm strainer. Cardiomyocytes were collected after two further rounds of gravity‐assisted settling, each lasting 20 min. All buffers were prepared fresh and stored at 37 °C before use. The isolated cardiomyocytes were stained with anti‐α‐actinin (1:100 dilution, Abcam, cat #ab9465) and DAPI (1:5000) for the nucleation analysis.

### Aden Associated Virus 9 (AAV9) Infection

The viruses used for infection experiments were constructed by OBiO Technology (ShangHai, China). For AAV9 infection of isolated neonatal mouse cardiomyocytes in vitro, cardiomyocytes were infected with 300 MOIs of AAV9‐*Klf1* or AAV9‐NC in DMEM/F12 medium as previously described.^[^ ^25^ ^]^ Additionally, the cardiomyocytes were treated with or without the inhibitory agent MSAB (MCE, 5 µM) according to previously study. The cardiomyocytes were harvested for RT‐qPCR or Western blot analysis 48 h after infection. EdU (Beyotime) was introduced into each culture medium at a dose of 10 µM according to the manufacturer's instructions at 48 h before harvest. The cardiomyocytes were stained for markers or interest after fixation with 4% paraformaldehyde. For AAV9 infection treatment in mice after myocardial infarction in vivo, each adult mouse was injected with 5×10^11^ vector genome (vg) virus through the tail vein at 1 DPI according to the manufacturer's instructions. The mice were examined via echocardiography at 28 DPI and then euthanized, after which the hearts were collected for Masson trichromatic staining and WGA staining.

### EdU Incorporation Assay

For isolated neonatal mouse cardiomyocytes cultured in vitro, 5‐ethynyl‐20‐deoxyuridine (EdU; Beyotime) was added to each culture at a concentration of 10 µM according to the manufacturer's instructions at 48 h before cardiomyocyte harvest. Cardiomyocytes were fixed with 4% paraformaldehyde prior to EdU tracing. For EdU labelling of proliferating cardiomyocytes in vivo, adult mice were intraperitoneally injected with EdU at 50 mg kg^−1^ body weight every 2 days for a total of five times before the heart was harvested according to previously described methods. For EdU immunofluorescence staining, rehydrated heart sections and fixed cells were incubated with BeyoClick EdU Cell Proliferation Kit (Beyotime) reagents for 30 min to assess EdU incorporation according to the manufacturer's instructions. Then, the sections were removed from the reaction mixture and rinsed three times with phosphate‐buffered saline (PBS). Cardiomyocytes were incubated with a mouse anti‐cardiac troponin T (Abcam, cat# ab8295, 1:100 dilution) primary antibody. Nuclei were visualized with 4′,6′‐diamidino‐phenylindole (DAPI, Servicebio).

### Echocardiography

Cardiac function was determined by echocardiography as previously described. Echocardiographic measurements were performed on the mice using an ultrasound and photoacoustic Vevo LAZR‐X Imaging System (FUJIFILM Visual Sonics) with a high‐frequency 40 MHz transducer (MX550D). The mice were anesthetized via inhalation of 2.5% isoflurane for induction and 0.5% isoflurane for maintenance. The murine heart rate should exceed 450 beats per minute to approximate the physiological state according to the established guidelines for evaluating cardiac physiology in mice. After alignment in the transverse B‐mode at the level of the papillary muscles, the cardiac values, including the left ventricular internal diameter at end‐diastole (LVEDd) and end‐systole (LVESd), were measured from the 2‐D short‐axis under M‐mode tracings. Left ventricular fractional shortening (FS) and ejection fraction (EF) values were calculated using the above primary measurements with the accompanying VevoLAB Version 3.2.0 software package (FUJIFILM Visual Sonics).

### Histology Analysis

To assess the histological profile and scar area, harvested hearts were rinsed and fixed in 4% paraformaldehyde at room temperature for 24 h. Fixed hearts were sequentially dehydrated in a series of graded ethanol solutions, cleared with xylene and finally embedded in paraffin according to standard laboratory procedures. To observe morphology, the embedded paraffin blocks were sectioned longitudinally along the four‐chamber cardiac incision plane for subsequent hematoxylin and eosin (H&E) staining. To estimate the scar area, the embedded hearts were continuously sliced latitudinally from the ligation site to the apex with an interval of 200 mm between each section (5 µm thick). Five sections collected from each heart underwent deparaffinization and rehydration and were subjected to Masson's trichrome staining. The scar area was calculated with ImageJ software by quantifying the percentage of scar tissue area (blue) relative to the total left ventricular area (red plus blue) as previously described. H&E and Masson's trichrome staining were performed using standard procedures as previously described.

### Immunofluorescence Analysis

To stain heart tissue with EdU and for proliferation markers of interest, paraffin‐embedded samples were cut into 5 µm sections, dewaxed in xylene for 20 min, and then successively rehydrated with decreasing concentrations of alcohol (100%, 95%, 85%, 75%, 50%) at room temperature. The sections were boiled in antigen retrieval buffer for 10 min, rinsed three times in PBS, permeabilized and blocked with PBS containing 0.3% Triton X‐100 and 3% bovine serum albumin (BSA) for 1 h at room temperature. EdU immunofluorescence staining was performed on the sections by using a BeyoClick EdU Cell Proliferation Kit (Beyotime) to assess EdU incorporation according to the manufacturer's instructions. For immunofluorescence of cardiomyocyte proliferation markers, the sections were then incubated with primary antibodies diluted in PBS containing 3% BSA at 4 °C overnight. After being washed with PBS three times, the sections were costained with isotype‐matched secondary antibodies for 1 h at room temperature, followed by staining with 2 µg mL^−1^ DAPI for 10 min in the dark. For immunofluorescence staining of cultured neonatal mouse cardiomyocytes, cells were fixed with 4% paraformaldehyde for 15 min at room temperature. The fixed cardiomyocytes were then permeabilized and blocked, followed by the same staining steps as those used for the heart sections. The primary antibodies used were as follows: anti‐α‐actinin (Abcam, cat#ab9465, 1:100 dilution), anti‐cardiac troponin T (Abcam, cat#ab8295, 1:100 dilution), anti‐KLF1 (Invitrogen, cat#PA5‐86441, 1:100 dilution), anti‐phospho‐histone H3 (CST, cat#3377, 1:100 dilution), anti‐Ki‐67 (Abcam, cat#ab16667, 1:100 dilution), and anti‐Aurora B (Abcam cat#ab2254, 1:100 dilution). Secondary antibodies conjugated to donkey anti‐rabbit Alexa Fluor 488 (Invitrogen, cat# A‐21206, 1:500 dilution) and donkey anti‐mouse Alexa Fluor 594 (Invitrogen, cat# A‐21203, 1:500 dilution) were used. Fluorescence was observed under an OLYMPUS FV3000 confocal laser scanning microscope (Olympus Corporation, Japan).

### WGA Staining and Cardiomyocyte Size Quantification

WGA staining and cardiomyocyte size quantification were performed as previously described. In brief, deparaffinization, antigen retrieval, and blocking of nonspecific background staining were routinely performed. The sections were then incubated with 10 µg mL^−1^ WGA conjugated to Alexa Fluor 647 (Invitrogen, cat# W32466) for 1 h at room temperature. Cardiomyocytes were stained with a mouse anti‐cardiac troponin T primary antibody. Nuclei were visualized with DAPI. Three different views of each heart from the left ventricle, right ventricle, and septum were captured. ImageJ was used to quantify the cross‐sectional size along the cardiomyocyte borders identified by WGA staining.

### Western Blot Analysis

Harvested heart or cardiomyocyte samples were homogenized and lysed in RIPA lysis buffer containing protease (Sigma, P8340, 1:100 dilution) and phosphatase inhibitor cocktails (Sigma, P5726 and P0044, 1:100 dilution) on ice. The protein concentration was measured via a BCA protein assay kit (Beyotime). Total protein was separated via 10% SDS‐PAGE and electrophoretically transferred onto PVDF membranes (Millipore). After nonspecific background staining was blocked with 5% skim milk at room temperature for 1 h, the membranes were incubated with primary antibodies at 4 °C overnight. The next day, the membranes were washed with Tris‐buffered saline (TBS) containing 0.1% Tween 20 and incubated with an isotype‐matched secondary antibody for 1 h at room temperature. The primary antibodies used were as follows: anti‐KLF1 (Invitrogen, cat# PA5‐86441), anti‐β‐catenin (Proteintech, cat# 17 565), anti‐c‐Myc (Proteintech, cat# 10 828), anti‐phospho‐β‐catenin (Proteintech, cat# 28 853), anti‐Cyclin D1 (Proteintech, cat# 26 939), and anti‐β‐actin (Proteintech, cat# 66 009). The blot signals were visualized using Pierce™ ECL Western blot Substrate (Thermo Fisher Scientific, 32 209). The abundance of each target protein was quantified via ImageJ software (NIH). Protein expression was normalized to that of β‐actin.

### Quantitative Real‐Time Polymerase Chain Reaction (qRT‐PCR)

To quantify gene expression, total RNA from heart tissues or cardiomyocyte samples was extracted with an RNeasy Mini Kit (QIAGEN, 74 004) according to the manufacturer's instructions. A Prime Script RT master mix kit (ABclonal, RK20402) was subsequently used to perform reverse transcription to synthesize single‐stranded complementary DNA (cDNA). qRT‒PCR was run on a Step One Plus Real‐time PCR system (Applied Biosystems, Thermo Fisher Scientific) with the addition of SYBR Premix Ex Taq (ABclonal, RK21207). The relative genome copy number was assessed via the comparative ΔΔCT method. Glyceraldehyde‐3‐phosphate dehydrogenase (*Gapdh*) or β‐actin was used as an internal control to normalize the expression of target RNA genes. Primers were listed in Table S1 (Supporting Information).

### RNA Sequencing and Data Analysis RNA Sequencing (RNA‐Seq)

Experiments were performed by OE Biotech (Shanghai, China). Briefly, total RNA was extracted from harvested hearts using a RNeasy kit (QIAGEN, 74 004). A Nanodrop 2000 and Agilent Bioanalyzer 2100 were used to assess the quantity and quality of total RNA, respectively. Total RNA (3 µg) with an RNA integrity number (RIN) of no less than 8.0 from each sample was used for library construction with a VAHTS Universal V6 RNA‐seq Library Prep Kit according to the manufacturer's instructions. The RNA‐seq libraries were sequenced via a NovaSeq 6000 system with 150‐cycle paired‐end reads. Raw reads were generated, and quality control was performed via standard protocols. Clean reads were aligned to the mm10 (UCSC) mouse genome with Subjunc (v2.0.1) using default parameters. The gene expression level was quantified as fragments per kilobase of exon per million fragments (FPKM) using featureCounts (v2.0.1). Differentially expressed genes were analyzed using the DESeq2 package (v1.30.1). Genes with a false discovery rate (*p*‐adjusted < 0.05) of less than five percent and with an absolute log2 (fold change) > 1 or 0.5 were chosen for downstream analysis and subsequent functional analyses. Volcano plots were plotted in R with ggplot2. Heatmaps were plotted in GENE‐E (Broad Institute). Gene Ontology (GO) enrichment analysis and statistical enrichment of differentially expressed genes in KEGG pathways were implemented via the R package ClusterProfiler (v4.3.0.991). Gene set enrichment analysis (GSEA) was performed using GSEA software.

### ATAC Sequencing and Data Analysis

ATAC sequencing (ATAC‐seq) was performed by SeqHealth technology (Wuhan, China). Briefly, 500 mg of heart tissue was lysed, and the nuclei were collected via standard protocols. Transposition and high‐throughput DNA sequencing libraries were prepared with a TruePrep DNA Library Prep Kit V2 for Illumina (Vazyme, cat# TD501) according to the manufacturer's instructions. After enrichment and quantification, the prepared libraries were sequenced on a NovaSeq 6000 sequencer (Illumina), and 150‐bp paired‐end reads were generated. The raw data were filtered via fastp (v0.23.1), and low‐quality reads contaminated by adaptor sequences were trimmed. Clean reads were then mapped to the reference mouse genome via Bowtie2 (v2.2.6) using default parameters. Sambamba (v0.7.1) was used for sam/bam format conversion and PCR duplicate read removal. The distribution of reads upstream and downstream of the TSS was visualized using DeepTools (v2.4.1). Read distribution analysis was performed with RSeQC (v2.6). Motif analysis was performed with Homer (v4.10). The different peaks were identified via csaw (v1.24.3). Annotated genes with absolute log2 (fold change) > 0.5 in both ATAC‐seq and RNA‐seq were screened for further Kyoto Encyclopedia of Genes and Genomes (KEGG) enrichment analysis.

### Statistical Analysis

All the data are expressed as means ± Standard Deviation (SD). Statistical analysis was performed using GraphPad Prism 9 software. Student's unpaired t test was used to assess significant differences between two groups. When more than two groups were compared, one‐way ANOVA was applied for analysis of variance. Log‐rank test was used for Kaplan‐Meier survival analysis. Results with *p* adjusted < 0.05 were considered statistically significant. Specific number of samples, individual *p* adjusted and statistical methods are detailed annotations in the Figure Legends.

## Conflict of Interest

The authors declare no conflict of interest.

## Author Contributions

Y. H., X. Z., and S. R. contributed equally to this work. J.W., J. X., and Z. Y. conceived, designed, and supervised the experiments. Y. H., X. Z., S. R., Y. L, W. Y., S. W., X. L., Z. L., J. Z., J. Z., K. Z., R. L., H. Z., L. L., P. H., Z. Z., and W. Z. performed the experiment. Y. H., X. Z., and S. R. analyzed the data and prepared figures. Y. H., X. Z., S. R., J.W., J. X., and Z. Y. wrote the final manuscript. Z. Y., X. Z., J. X., and J.W. provided the financial support.

## Supporting information



Supporting Information

## Data Availability

The data that support the findings of this study are available from the corresponding author upon reasonable request.
