# Supplementary material for: KLF1 Promotes Cardiomyocyte Proliferation and Heart Regeneration Through Regulation of Wnt/β‐Catenin Signaling Pathway
Source: Adv Sci (Weinh). 2025 Mar 27;12(21):2413964. doi: 10.1002/advs.202413964 (PMC12140325; doi:10.1002/advs.202413964)

Supporting Information

KLF1 Promotes Cardiomyocyte Proliferation and Heart Regeneration through Regulation of Wnt/β-catenin Signaling Pathway

Yanglin Hao^#^, Xi Zhang^#^, Shuan Ran^#^, Yuan Li, Weicong Ye, Song Wang, Xiaohan Li, Zilong Luo, Jiulu Zhao, Junjie Zong, Kexiao Zheng, Ran Li, Han Zhang, Longyong Lai, Pinyan Huang, Zifeng Zou, Wang Zhan, Zhang Yue*, Jie Wu*, Jiahong Xia*

Y. Hao, X. Zhang, S. Ran, Y. Li, W. Ye, S. Wang, X. Li, Z. Luo, J. Zhao, J. Zong, K. Zheng, R. Li, H. Zhang, L. Lai, P. Huang, Z. Zou, W. Zhan, Z. Yue, J. Wu, J. Xia

Department of Cardiovascular Surgery

Union Hospital

Tongji Medical College

Huazhong University of Science and Technology

Wuhan 430022, China

**#These authors contribute equally.**

E-mail: [jiahong.xia@hust.edu.cn](mailto:jiahong.xia@mail.hust.edu.cn); [wujie426@hust.edu.cn](mailto:wujie426@hust.edu.cn); yuegaoxin1120@ hust.edu.cn

**Figure S1**

**
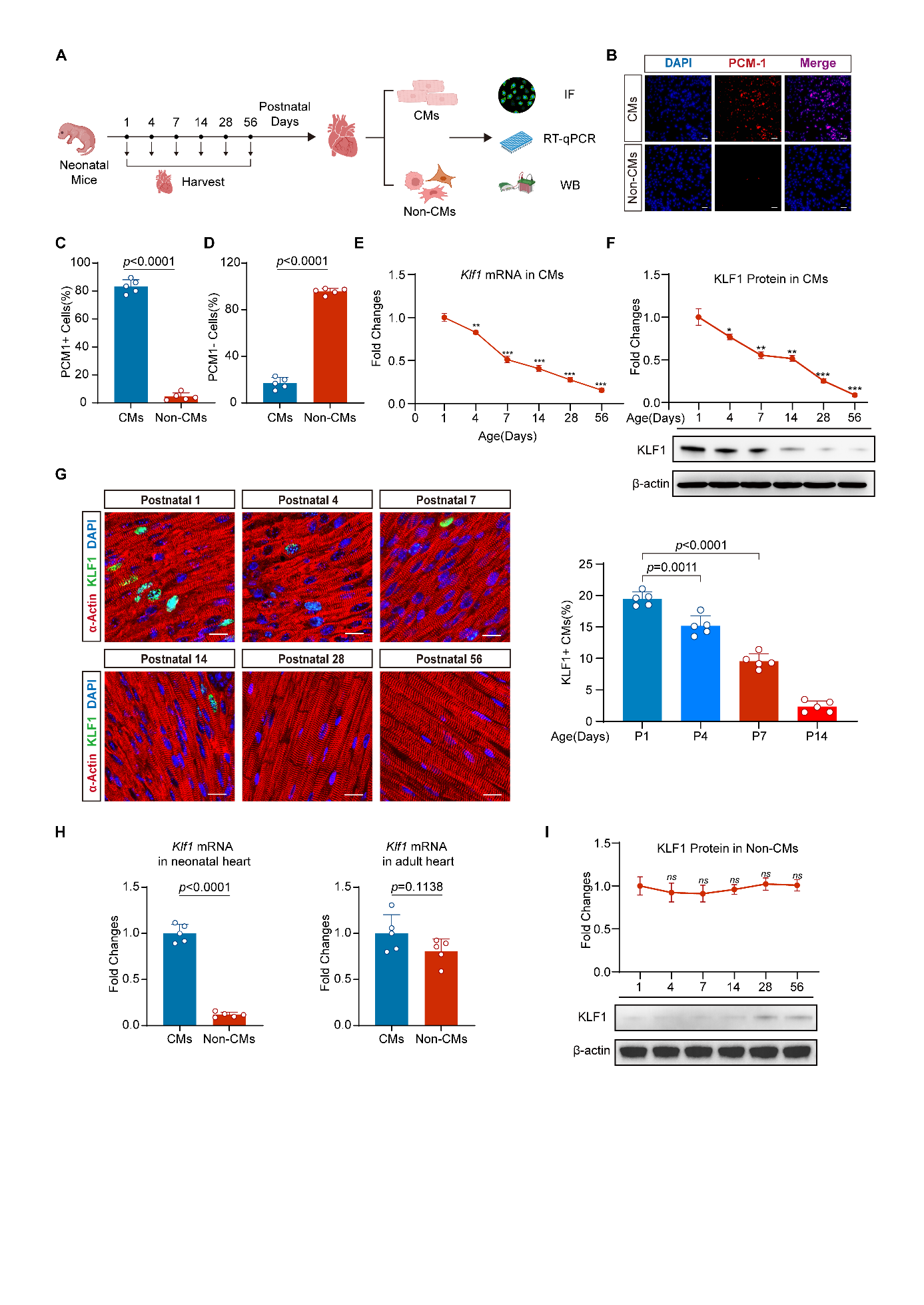
**

**Figure S1. Expression of KLF1 in the mouse heart at steady state.**

**A,** Schematic of the experimental design: WT C57BL/6 hearts were collected at representative postnatal timepoints. Cardiomyocytes and non-cardiomyocytes in the hearts were then separated according to the differential time adherence method or a Langendorff-free perfusion method for subsequent experiments. **B,** Representative immunofluorescence images of cardiomyocytes and non-cardiomyocytes isolated from P1 neonatal hearts stained with DAPI and PCM-1 (PCM-1, red; DAPI, blue). Scale bar, 20 µm. **C and D,** The bar graphs show the percentages of PCM-1^+^ (C) and PCM-1^-^ (D) cells in isolated cardiomyocytes and non-cardiomyocytes (n = 5; 2-tailed unpaired Student’s t test).  **E and F,** The mRNA (E) and protein (F) levels of KLF1 at representative time points (P1, P4, P7, P14, P28, and P56) in isolated cardiomyocytes of WT hearts at steady state were measured by PCR and Western blot (n = 3; one-way ANOVA). **G,** Representative confocal microscopy images of heart sections from WT mice at representative time points (P1, P4, P7, P14, P28, and P56), (a-Actin, red; KLF1, green; and DAPI, blue). Scale bar, 15 µm. The bar graph shows the percentages of KLF1^+^ cardiomyocytes (n = 5; one-way ANOVA). **H,** The mRNA levels of *Klf1* in isolated cardiomyocytes and non-cardiomyocytes of P1 neonatal (left) and P56 adult (right) hearts at steady state were measured by PCR (n = 5; 2-tailed unpaired Student’s t test). **I,** The protein levels of KLF1 at representative time points (P1, P4, P7, P14, P28, and P56) in isolated non-cardiomyocytes of WT hearts at steady state were measured by Western blot (n = 3; one-way ANOVA). IF, immunofluorescence; WB, Western blot.

**Figure S2**

**
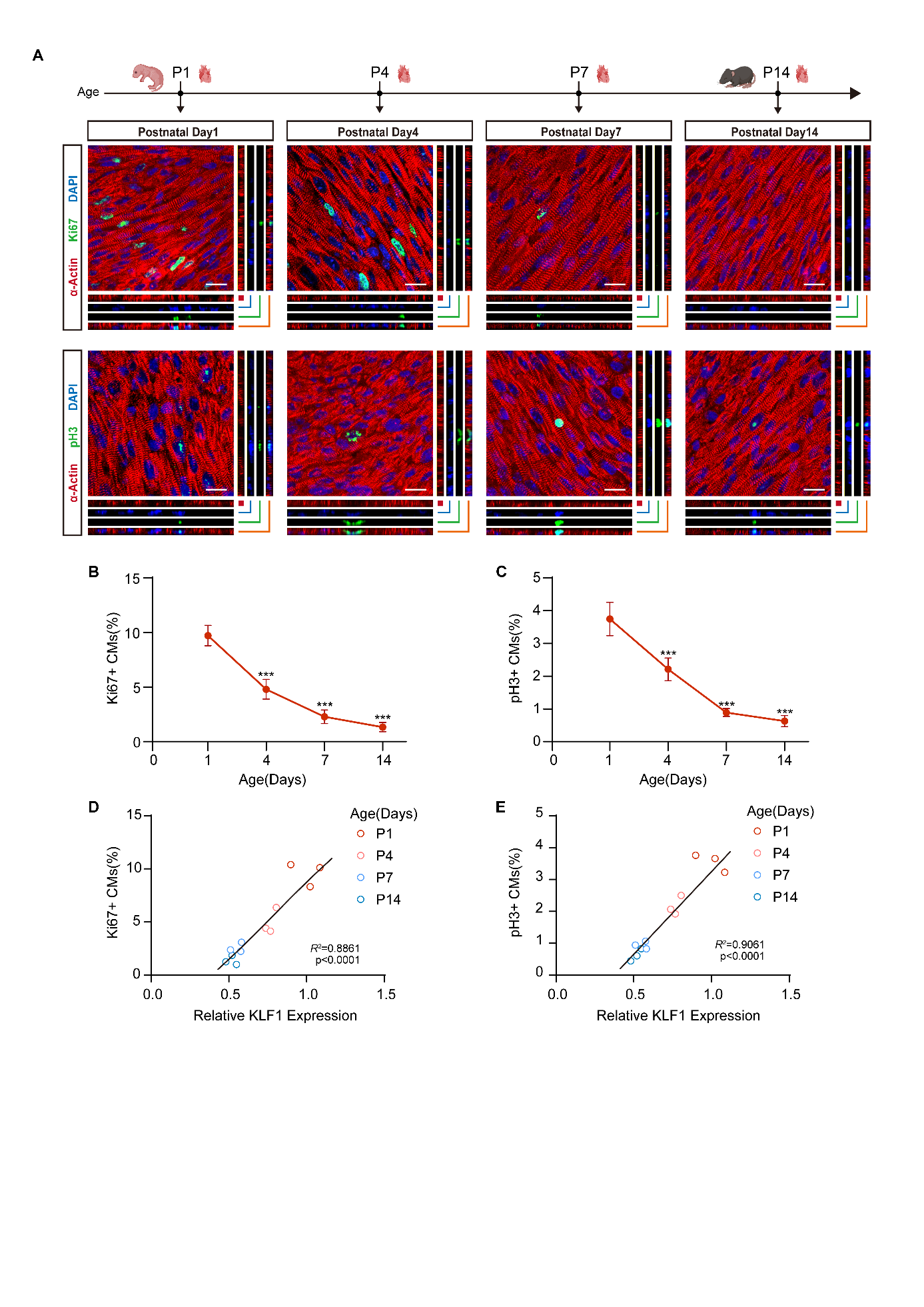
**

**Figure S2. Correlation analysis between KLF1 expression and cardiomyocyte proliferation.**

**A,** Representative confocal microscopy images of heart sections from P1, P4, P7, and P14 WT mice at steady state (a-Actin, red; Ki-67 and pH3, green; and DAPI, blue). Scale bar, 15 µm. **B and C,** Line graph shows the percentage of Ki-67^+^ (B) and pH3^+^ (C) cardiomyocytes in heart sections of WT mice at representative time points in the steady state (n = 5; one-way ANOVA). **D and E,** Correlation analysis of cardiomyocyte KLF1 protein expression levels with the percentage of Ki-67^+^ (D) and pH3^+^ (E) cardiomyocytes in the hearts of WT mice at representative time points in the steady state.

**Figure S3**


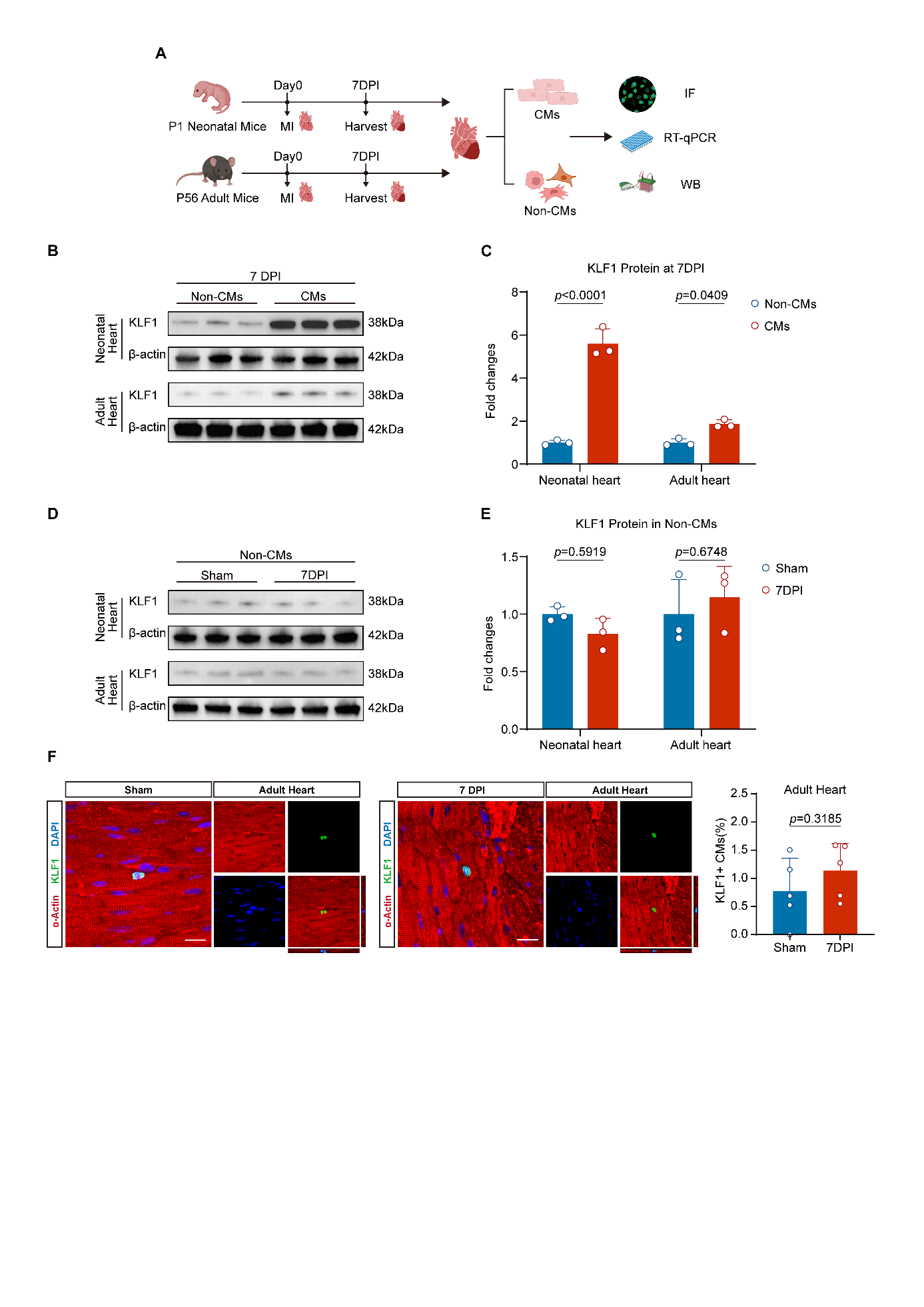


**Figure S3: KLF1 is mainly expressed in cardiomyocytes rather than non-cardiomyocytes.**

**A,** Schematic of the experimental design: MI was induced in P1 neonatal mice and P56 adult mice, and the hearts were harvested at 7 DPI. Cardiomyocytes and non-cardiomyocytes in the hearts were then separated according to the differential time adherence method or a Langendorff-free perfusion method for subsequent experiments. **B,** The protein levels of KLF1 in isolated non-cardiomyocytes and cardiomyocytes of neonatal and adult hearts at 7 DPI were measured via Western blot. **C,** Bar graph shows the quantification of KLF1 protein levels in non-cardiomyocytes and cardiomyocytes of neonatal and adult hearts in Figure S3B (n = 3; two-way ANOVA). **D,** The protein levels of KLF1 in non-cardiomyocytes of neonatal and adult hearts at 7 DPI were measured via Western blot. **E,** Bar graph shows the quantification of KLF1 protein levels in non-cardiomyocytes of neonatal and adult hearts in Figure S3D (n = 3; two-way ANOVA). **F,** Representative confocal microscopy images of heart sections from adult WT mice at 7 DPI (a-Actin, red; KLF1, green; and DAPI, blue). Scale bar, 15 µm. The bar graph shows the percentage of KLF1^+^ cardiomyocytes (n = 5; 2-tailed unpaired Student’s t test).

**Figure S4**


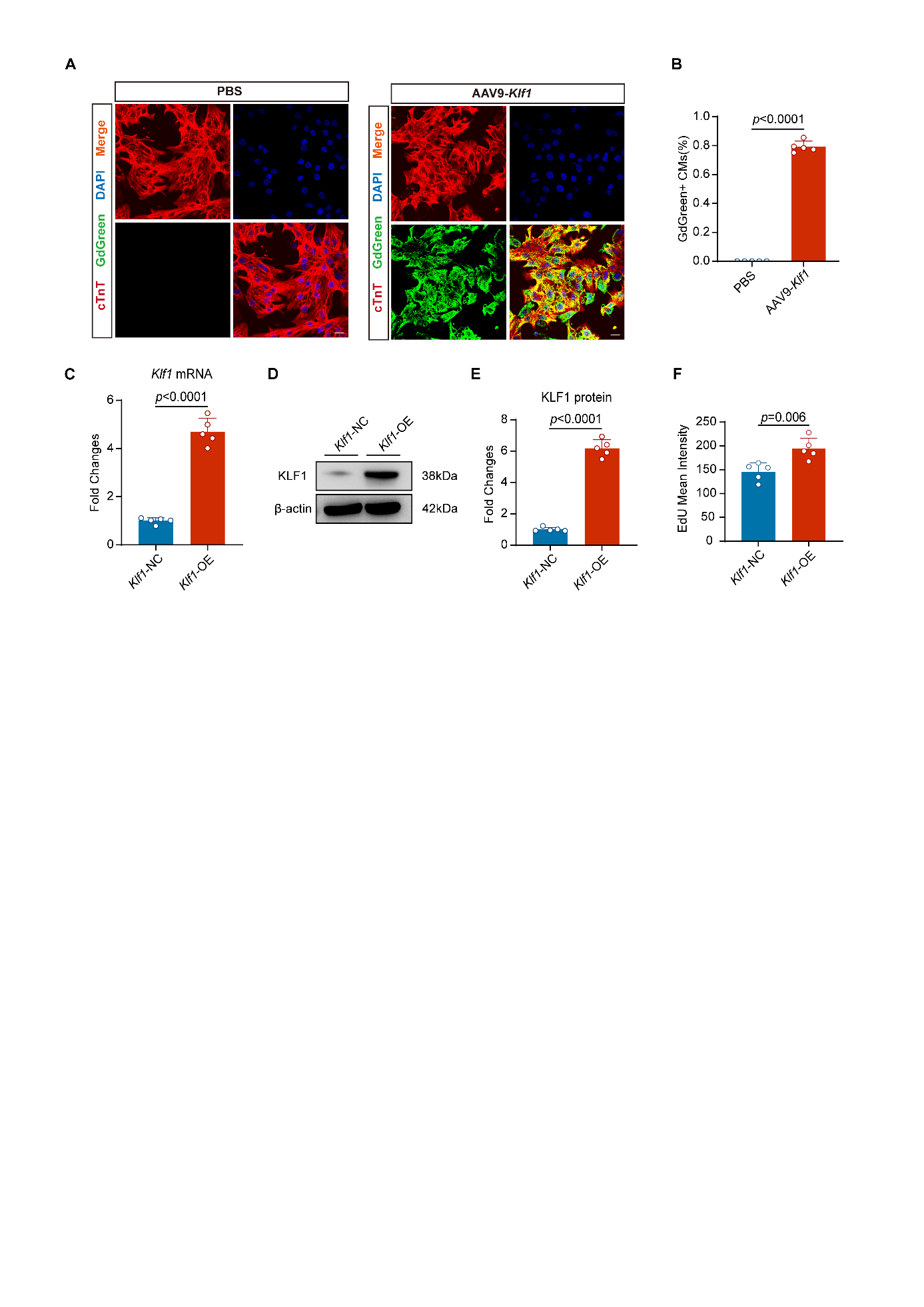


**Figure S4: AAV9-*Klf1* was used to overexpress KLF1 in isolated neonatal mouse CMs *in vitro*.**

**A,** Representative confocal microscopy images of isolated cardiomyocytes from postnatal day 7 (P7) mice transduced with AAV9-*Klf1* for 48 h (cTnT, red; GdGreen, green; and DAPI, blue). Scale bar, 20 µm. **B,** The bar graph shows the percentages of GdGreen^+^ cardiomyocytes (n = 5; 2-tailed unpaired Student’s t test). **C,** The mRNA levels of *Klf1* in isolated P7 cardiomyocytes transduced with AAV9-*Klf1* for 48 h were measured by PCR (n = 5; 2-tailed unpaired Student’s t test). **D,** The protein levels of KLF1 in isolated P7 cardiomyocytes transduced with AAV9-*Klf1* for 48 h were measured by Western blot. **E,** Bar graph shows the quantification of KLF1 protein levels in isolated P7 cardiomyocytes transduced with AAV9-*Klf1* for 48 h measured by Western blot in Figure S4D (n = 5; 2-tailed unpaired Student’s t test). **F,** Bar graph shows the intensity of EdU-stained cardiomyocytes among isolated P7 cardiomyocytes transduced with AAV9-*Klf1* for 48 h (n = 5; 2-tailed unpaired Student’s t test).

**Figure S5**

**
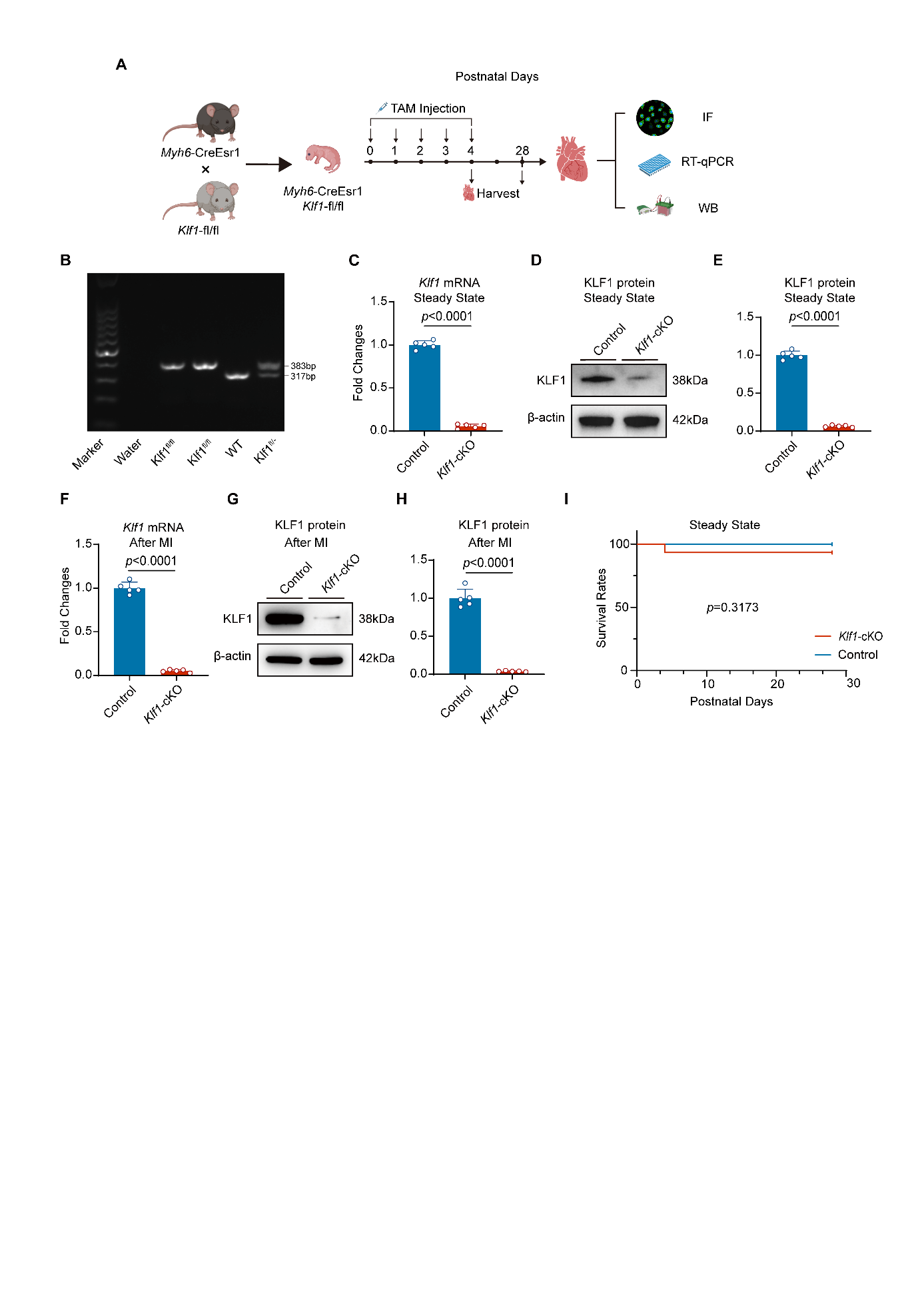
**

**Figure S5. Generation and verification of *Klf1*-cKO mice.**

**A,** Schematic of the experimental design: *Klf1*-cKO mice were generated by crossing Myh6-CreEsr1 mice with *Klf1*-fl/fl mice, and *Klf1* knockout was induced by continuous tamoxifen injection for five days after birth. The hearts were harvested at P4 and P28 for subsequent experiments. **B,** To confirm the genotypes of the homozygous *Klf1*-cKO mice, tail biopsies were collected, and genotype identification was performed via RT‒qPCR and agarose gel electrophoresis. **C,** The mRNA levels of *Klf1* in the hearts of P4 neonatal *Klf1*-cKO mice with TAM injection in steady state were measured via RT-qPCR (n = 5; 2-tailed unpaired Student’s t test). **D,** The protein levels of KLF1 in the hearts of P4 neonatal *Klf1*-cKO mice with TAM injection in steady state were measured via Western blot. **E,** Bar graph shows the quantification of KLF1 protein levels in the hearts of P4 neonatal *Klf1*-cKO mice measured by Western blot in Figure S5D (n = 5; 2-tailed unpaired Student’s t test). **F,** The mRNA levels of *Klf1* in the hearts of neonatal *Klf1*-cKO mice treated with TAM were measured via RT-qPCR at 7DPI (n = 5; 2-tailed unpaired Student’s t test). **G,** The protein levels of KLF1 in the hearts of neonatal *Klf1*-cKO mice treated with TAM were measured via Western blot at 7DPI. **H,** Bar graph shows the quantification of KLF1 protein levels in the hearts of neonatal *Klf1*-cKO mice measured by Western blot in Figure S5G (n = 5; 2-tailed unpaired Student’s t test). **I,** Survival curves of *Klf1*-cKO and control mice during postnatal growth at steady state (n = 15; log-rank test). TAM, tamoxifen.

**Figure S6**

**
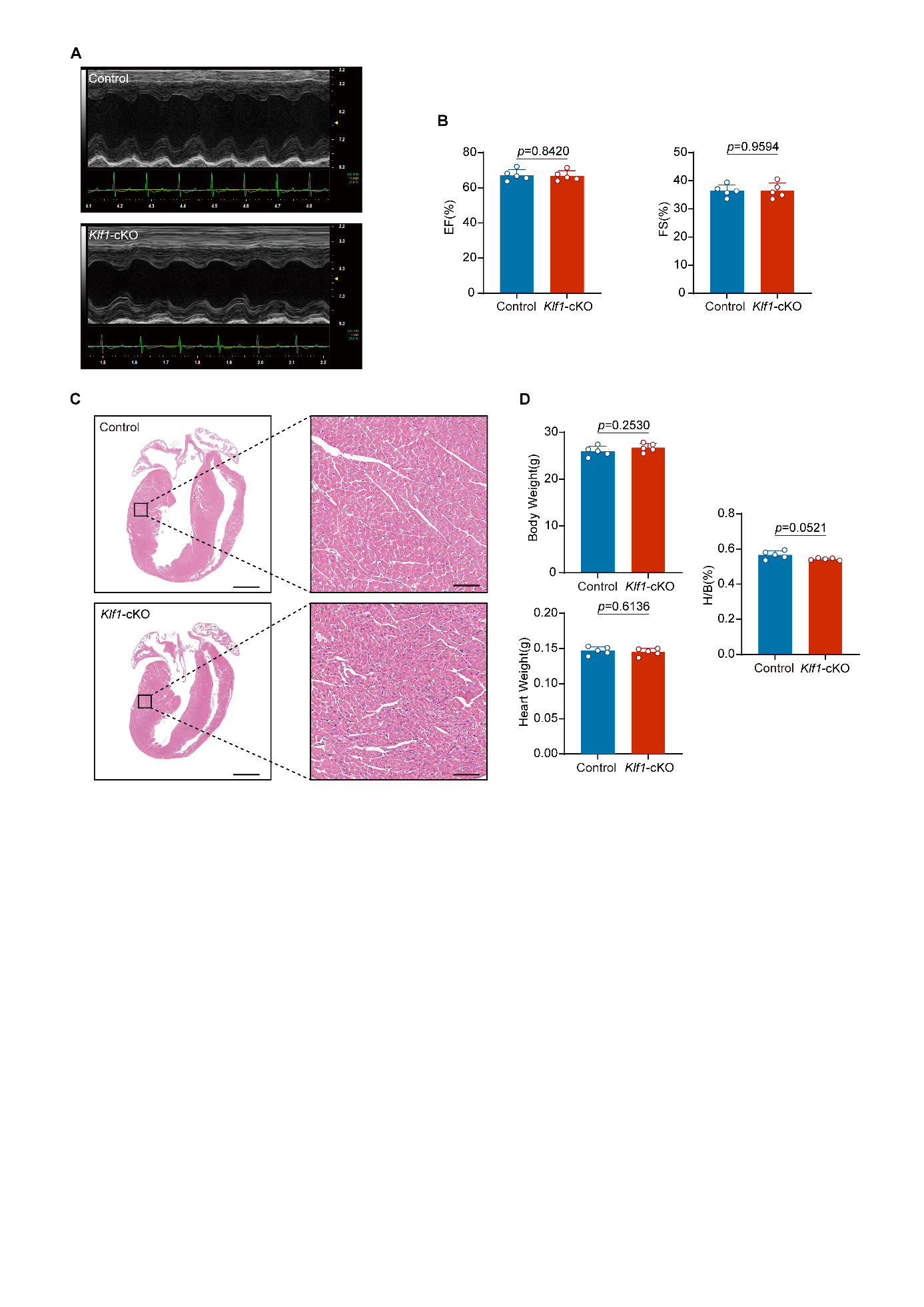
**

**Figure S6. Cardiac function and structure of *Klf1*-cKO mice at steady state.**

**A and B,** Representative M-mode echocardiography images of hearts from control and *Klf1*-cKO mice at steady state. The bar graph shows the EF and FS values for hearts from control and *Klf1*-cKO mice at steady state (n = 5; 2-tailed unpaired Student’s t test). **C,** Hematoxylin and eosin-stained images of hearts from P28 control and *Klf1*-cKO mice in the 4-chamber (left) and partially enlarged (right) views. Scale bar, 1 mm in the 4-chamber view (left); 100 µm in the partially enlarged view (right). **D,** Bar graph shows the body weight, heart weight and heart-to-body weight ratio in P28 control and *Klf1*-cKO mice at steady state (n = 5; 2-tailed unpaired Student’s t test).

**Figure S7**

**
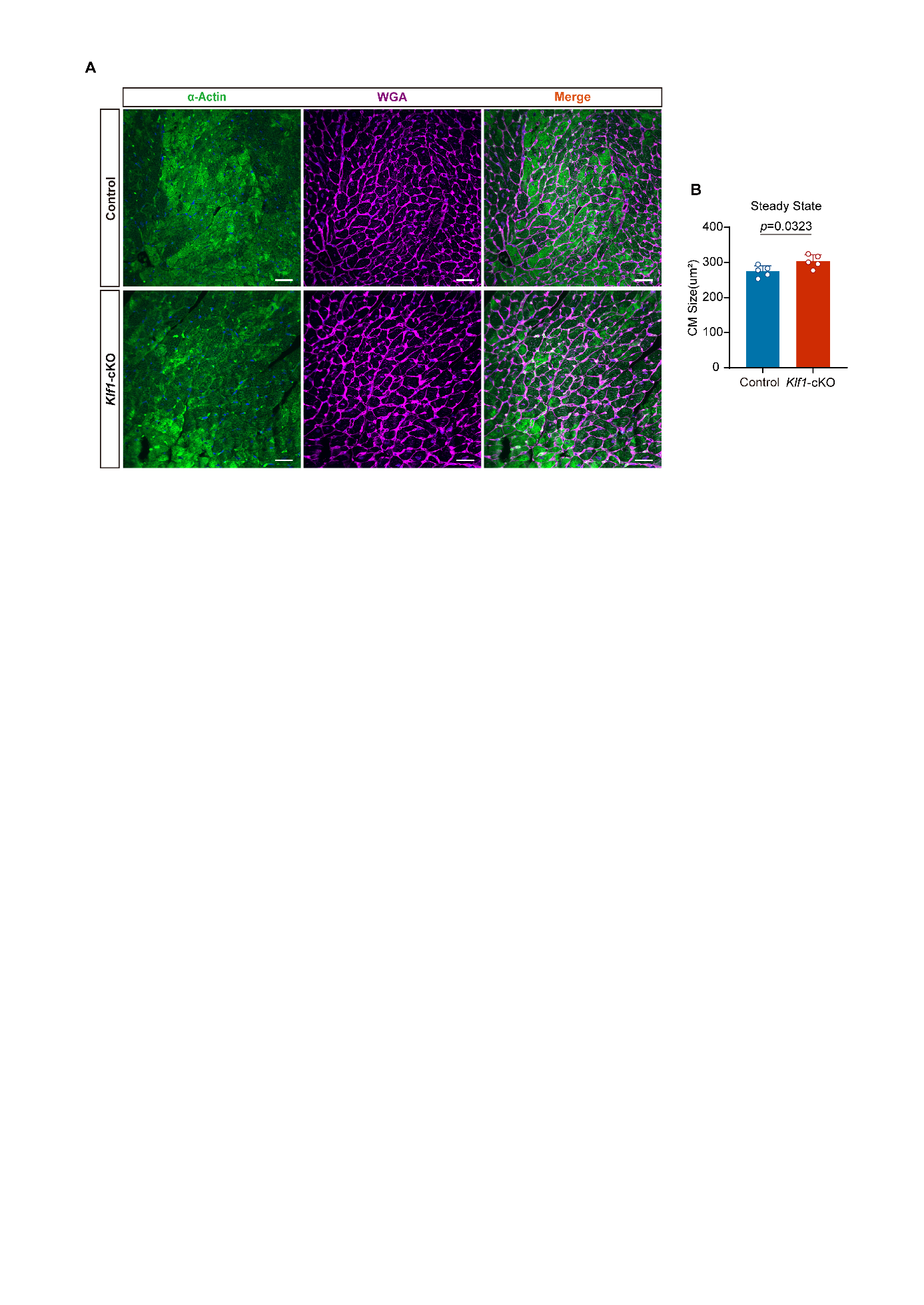
**

**Figure S7. Cardiomyocyte size was slightly increased in *Klf1*-cKO mice.**

**A****,** Representative WGA staining of heart sections from P28 control and *Klf1*-cKO mice at steady state (WGA, purple; a-Actin, green; and DAPI, blue). Scale bar, 20 µm. **B,** Bar graph shows the quantification of average cardiomyocyte size based on WGA staining at steady state in Figure S7A (n = 5; 2-tailed unpaired Student’s t test).

**Figure S8**


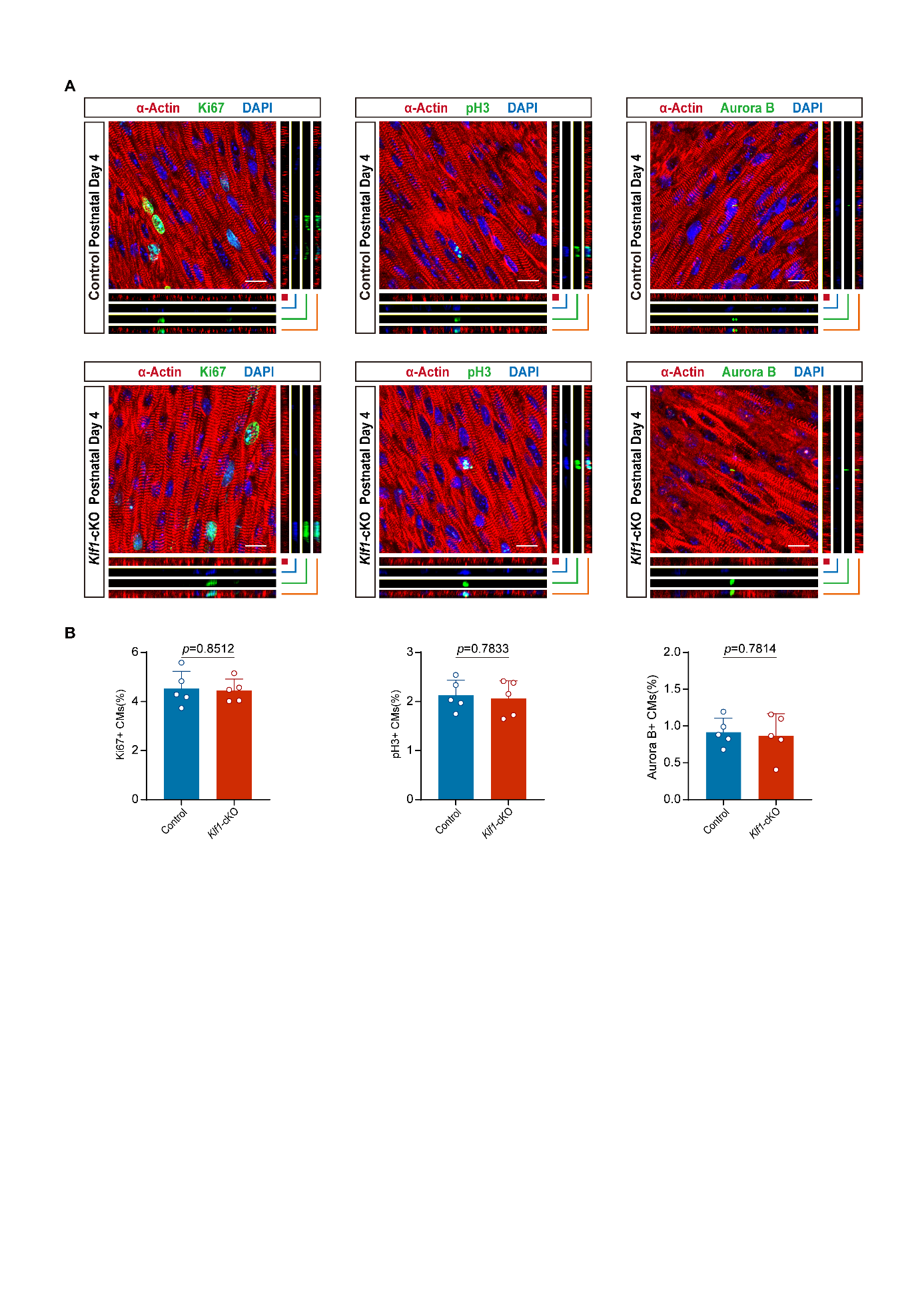


**Figure S8. *Klf1* knockout has a minimal effect on cardiomyocyte proliferation at steady state.**

**A,** Representative confocal microscopy images of heart sections from P4 control and *Klf1*-cKO mice at steady state (a-Actin, red; Ki-67, pH3, and Aurora B, green; and DAPI, blue). Scale bar, 15 µm. **B,** Bar graph shows the percentages of Ki-67^+^, pH3^+^, and Aurora B^+^ cardiomyocytes (n = 5; 2-tailed unpaired Student’s t test).

**Figure S9**

**
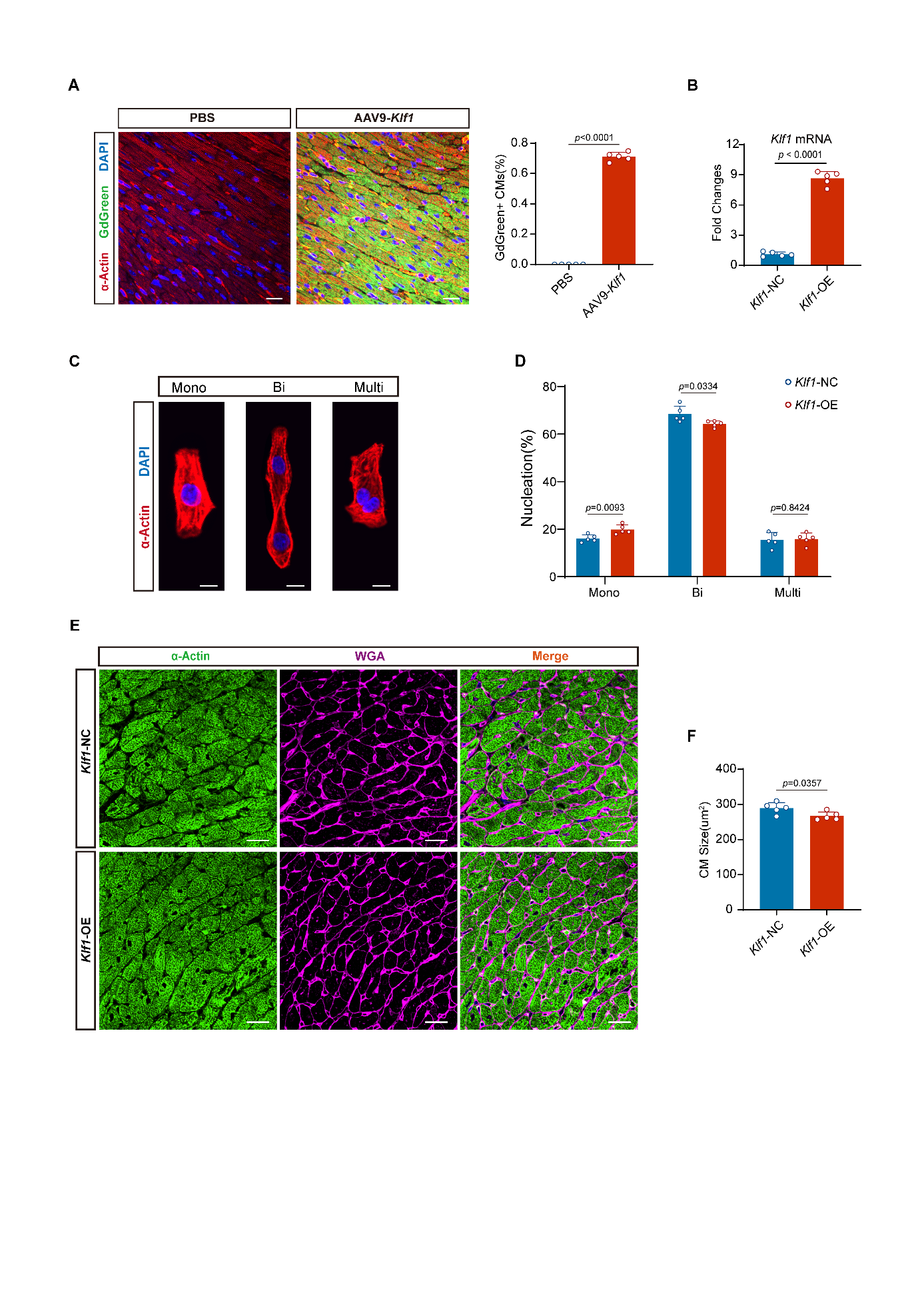
**

**Figure S9. Forced KLF1 overexpression increases cardiomyocyte proliferation and heart regeneration in adult mice after MI. A,** Representative confocal microscopy images of heart sections from *Klf1*-OE mice at 14 DPI (cTnT, red; GdGreen, green; and DAPI, blue). Scale bar, 20 µm. The bar graph shows the percentages of GdGreen^+^ cardiomyocytes (n = 5; 2-tailed unpaired Student’s t test). **B,** The mRNA levels of *Klf1* in the hearts of *Klf1*-OE mice were measured via RT‒qPCR at 14 DPI (n = 5; 2-tailed unpaired Student’s t test). **C,** Representative confocal microscopy images of isolated cardiomyocytes from *Klf1*-OE mice at 14 DPI (a-Actin, red; DAPI, blue). Scale bar, 20 µm. **D,** Bar graph shows the number of nuclei in *Klf1*-OE mice at 14 DPI (n = 5, multiple t test). Mono, mononuclear. Bi, binuclear. Multi, multinuclear. **E and F,** Representative WGA staining (E) of heart sections from *Klf1*-OE and *Klf1*-NC mice at 28 DPI (WGA, purple; a-Actin, red; and DAPI, blue). Scale bar, 20 µm. Bar graph (F) shows the average cardiomyocyte size based on WGA staining in Figure S9E (n = 5; 2-tailed unpaired Student’s t test).

**Figure S10**

**
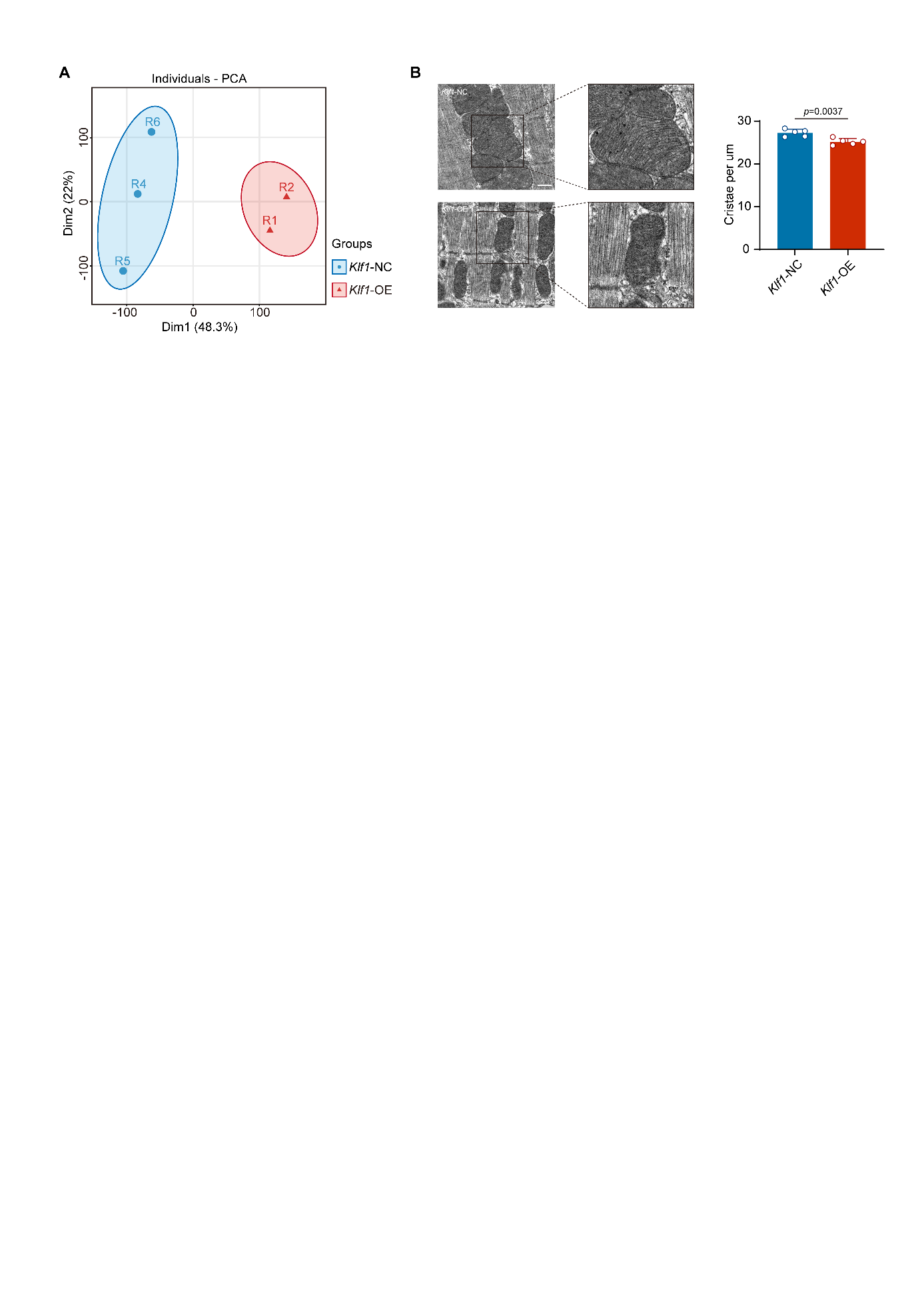
**

**Figure S10. Transcriptional reprogramming and mitochondrial structure alterations in the hearts of *Klf1*-OE mice.**

**A,** Principal component analysis (PCA) of *Klf1*-OE and *Klf1*-NC mouse transcriptomic data. The blue dots represent the hearts of *Klf1*-NC mice, and the red dots represent the hearts of *Klf1*-OE mice. The ellipses represent the confidence intervals of each group. **B,** Transmission electron microscopy (TEM) of the mitochondrial ultrastructure of the hearts of *Klf1*-OE and *Klf1*-NC mice at 14 DPI. Scale bar, 500nm. Bar graph shows the mean density of mitochondrial cristae (n = 5; 2-tailed unpaired Student’s t test).

**Tables**

**Table S1: Oligonucleotide** **sequences**

| **Mouse genotype primer sequences** | |
| --- | --- |
| *Klf1^flox/flox^* mice | AGGGGTCTGAGATCAAGGTGA |
|  | CGGTTCCCCTAACCCCTTTC |
| *Myh6-*CreEsr1  mice | CCAACTCTTGTGAGAGGAGCA |
|  | TCTATTGCACACAGCAATCCA |
|  | CCAGCATTGTGAGAACAAGG |
| **qRT-PCR primer sequences** | |
| *Klf1* | AGACTGTCTTACCCTCCATCAG |
|  | GGTCCTCCGATTTCAGACTCAC |
| *Actn* | GGCTGTATTCCCCTCCATCG |
|  | CCAGTTGGTAACAATGCCATGT |

**Table S2: Antibodies used in the experiments**

| **Antibodies** | **Source** | **Identifier** |
| --- | --- | --- |
| Mouse monoclonal to Cardiac Troponin T | Abcam | ab8295 |
| Mouse monoclonal to Sarcomeric Alpha Actinin | Abcam | ab9465 |
| Rabbit monoclonal Anti-Ki67 antibody | Abcam | ab16667 |
| Rabbit polyclonal Anti-Aurora B antibody | Abcam | ab2254 |
| Phospho-Histone H3 Rabbit monoclonal antibody | CST | 3377 |
| EKLF Polyclonal Antibody | Invitrogen | PA5-86441 |
| Alexa Fluor 488 conjugated Donkey anti-Rabbit IgG | Invitrogen | A-21206 |
| Alexa Fluor 594 conjugated Donkey anti-Mouse IgG | Invitrogen | A-21203 |
| Alexa Fluor 647 conjugated Donkey anti-Rabbit IgG | Invitrogen | A-31573 |
| Beta Catenin Polyclonal antibody | Proteintech | 17565 |
| c-MYC Polyclonal antibody | Proteintech | 10828 |
| Phospho-Beta Catenin (Ser675) Polyclonal antibody | Proteintech | 28853 |
| Cyclin D1 Polyclonal antibody | Proteintech | 26939 |
| Beta Actin Monoclonal antibody | Proteintech |  |
| HRP-conjugated Affinipure Goat Anti-Mouse IgG | Proteintech | SA00012-1 |
| HRP-conjugated Affinipure Goat Anti-Rabbit IgG | Proteintech | SA00001-2 |

**Unedited gel for each representative cropped gel within the manuscript:**


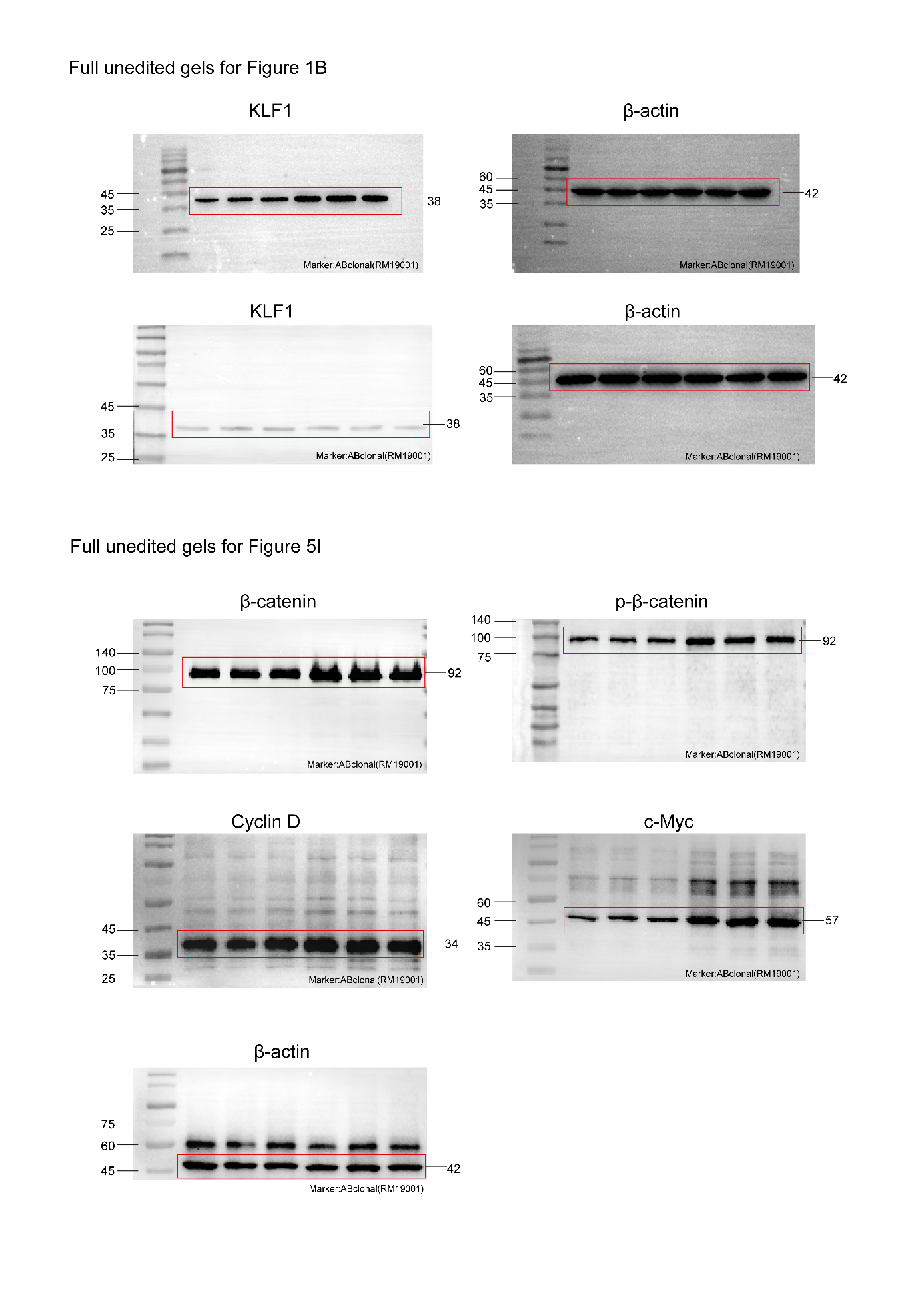


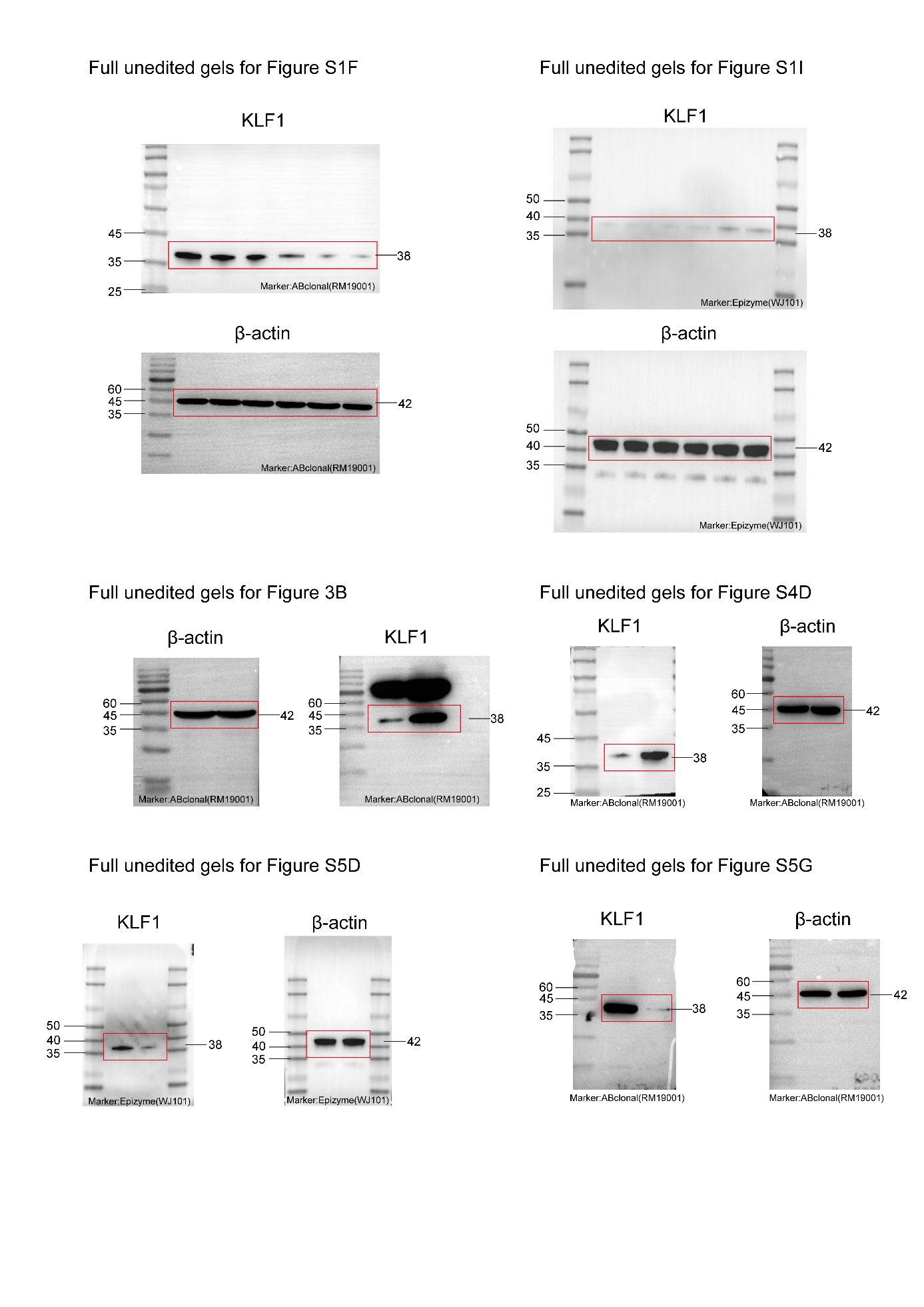

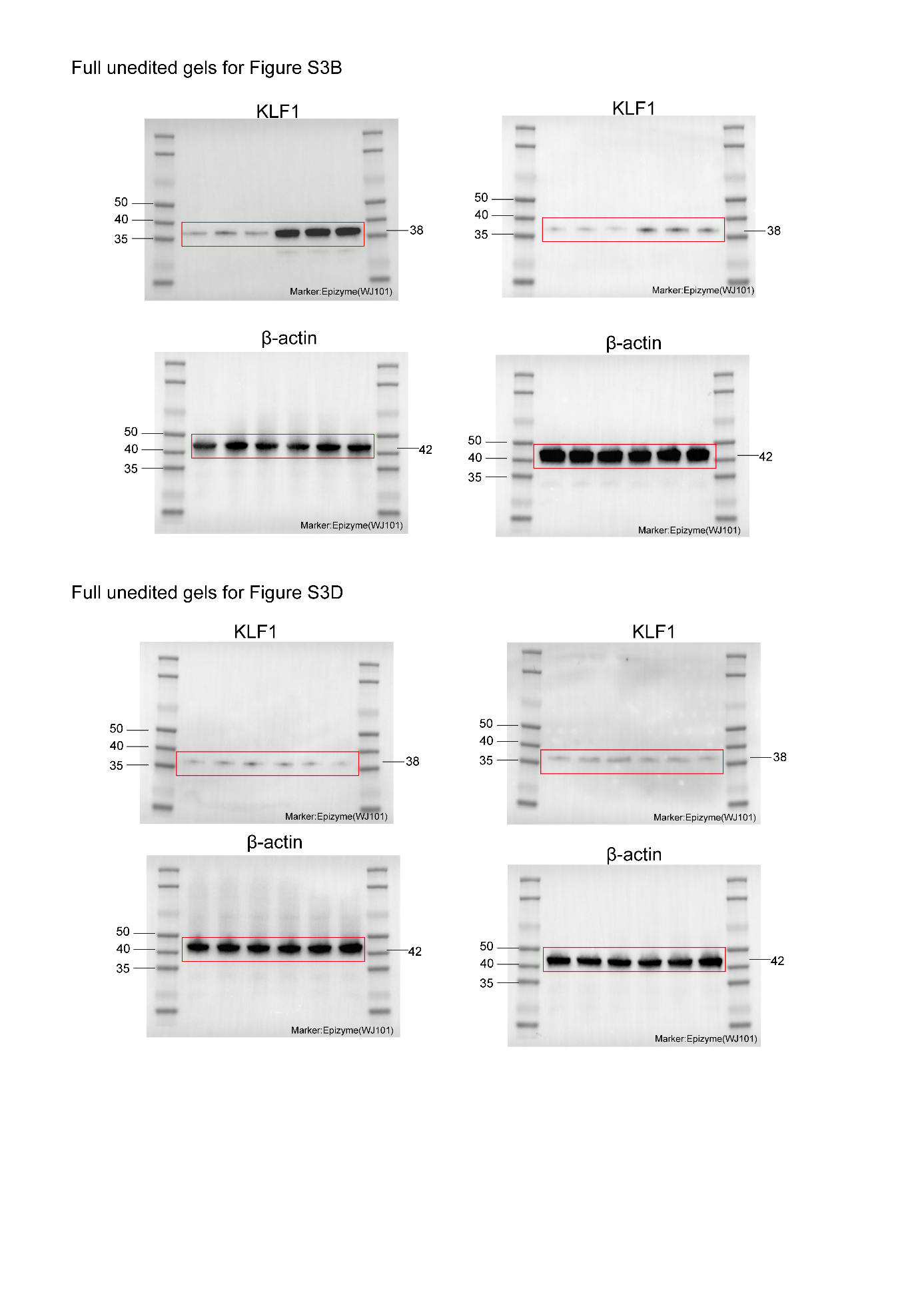

Supplement: Supplementary file 1 — Supporting Information [file ADVS-12-2413964-s001.docx]
